# A Recently Formed Triploid *Cardamine insueta* Inherits Leaf Vivipary and Submergence Tolerance Traits of Parents

**DOI:** 10.3389/fgene.2020.567262

**Published:** 2020-10-06

**Authors:** Jianqiang Sun, Rie Shimizu-Inatsugi, Hugo Hofhuis, Kentaro Shimizu, Angela Hay, Kentaro K. Shimizu, Jun Sese

**Affiliations:** ^1^Research Center for Agricultural Information Technology, National Agriculture and Food Research Organization, Tsukuba, Japan; ^2^Department of Evolutionary Biology and Environmental Studies, University of Zurich, Zurich, Switzerland; ^3^Max Planck Institute for Plant Breeding Research, Cologne, Germany; ^4^Department of Biotechnology, Graduate School of Agricultural and Life Sciences, The University of Tokyo, Tokyo, Japan; ^5^Kihara Institute for Biological Research (KIBR), Yokohama City University, Yokohama, Japan; ^6^Artificial Intelligence Research Center, National Institute of Advanced Industrial Science and Technology, Tokyo, Japan; ^7^Humanome Lab, Inc., Tokyo, Japan

**Keywords:** allopolyploid, homeolog, RNA-seq, meristem formation, ecological niche

## Abstract

Contemporary speciation provides a unique opportunity to directly observe the traits and environmental responses of a new species. *Cardamine insueta* is an allotriploid species that appeared within the past 150 years in a Swiss village, Urnerboden. In contrast to its two progenitor species, *Cardamine amara* and *Cardamine rivularis* that live in wet and open habitats, respectively, *C. insueta* is found in-between their habitats with temporal water level fluctuation. This triploid species propagates clonally and serves as a triploid bridge to form higher ploidy species. Although niche separation is observed in field studies, the mechanisms underlying the environmental robustness of *C. insueta* are not clear. To characterize responses to a fluctuating environment, we performed a time-course analysis of homeolog gene expression in *C. insueta* in response to submergence treatment. For this purpose, the two parental (*C. amara* and *C. rivularis*) genome sequences were assembled with a reference-guided approach, and homeolog-specific gene expression was quantified using HomeoRoq software. We found that *C. insueta* and *C. rivularis* initiated vegetative propagation by forming ectopic meristems on leaves, while *C. amara* did not. We examined homeolog-specific gene expression of three species at nine time points during the treatment. The genome-wide expression ratio of homeolog pairs was 2:1 over the time-course, consistent with the ploidy number. By searching the genes with high coefficient of variation of expression over time-course transcriptome data, we found many known key transcriptional factors related to meristem development and formation upregulated in both *C. rivularis* and *rivularis*-homeolog of *C. insueta*, but not in *C. amara*. Moreover, some *amara*-homeologs of these genes were also upregulated in the triploid, suggesting *trans*-regulation. In turn, Gene Ontology analysis suggested that the expression pattern of submergence tolerant genes in the triploid was inherited from *C. amara*. These results suggest that the triploid *C. insueta* combined advantageous patterns of parental transcriptomes to contribute to its establishment in a new niche along a water-usage gradient.

## Introduction

The molecular basis of speciation has been a central question in biology (Coyne and Orr, 2004). Little is known still about how a new species obtains new traits to adapt to a distinct environment. A major obstacle in studying this is that most speciation events occurred in the past, and thus the traits and the environment at the time of speciation are not directly observable. The difference in traits and environments between current species may represent evolution after speciation rather than the changes that occurred at speciation. A unique opportunity to study speciation in action is contemporary allopolyploid speciation (Soltis and Soltis, 2009; Abbott et al., 2013). Several cases of polyploid speciation during the past 150 years have been documented, for example in *Tragopogon*, *Senecio*, *Mimulus*, *Spartina*, and *Cardamine* (Urbanska et al., 1997; Abbott and Andrew, 2004; Ainouche et al., 2004; Soltis et al., 2004). Because polyploid speciation immediately confers complete or partial reproductive isolation between the new polyploid and progenitor species, a new polyploid species must establish and propagate while surrounded by individuals with different ploidy. To overcome this situation termed “minor cytotype disadvantage,” two traits are suggested to facilitate establishment (Comai, 2005). First, the distinct environmental niche of a polyploid species would reduce competition with progenitor species. Second, clonal vegetative propagation or self-fertilization would assure the persistence of new polyploids at the initial stages because meiotic abnormality is common in newly formed polyploid species (Levin, 2002; Comai, 2005; Cifuentes et al., 2010; Zielinski and Mittelsten Scheid, 2012). This would be critical for odd-ploidy species including triploids, which often contribute to the formation of higher polyploids via a so-called triploid bridge (Bretagnolle and Thompson, 1995; Ramsey and Schemske, 1998; Mable, 2003; Husband, 2004; Tayalé and Parisod, 2013; Mason and Pires, 2015). Despite the significance of these traits, the underlying molecular mechanisms are yet to be studied.

**FIGURE 1:**
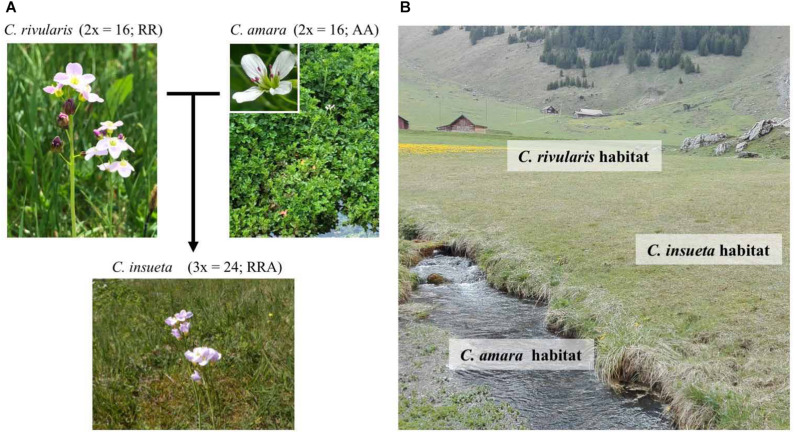
Habitats and relations among the three *Cardamine* species. **(A)** *C. insueta* (triploid) was naturally formed by hybridization between *C. amara* (diploid) and *C. rivularis* (diploid) 100–150 years ago in their natural habitat in Swiss Alps. **(B)** Conceptual indication of the habitats of three species at Urnerboden: *C. amara* prefers wet habitats along waterside; *C. rivularis* prefers meadow; and allotriploid *C. insueta* can be found between them.

The contemporary polyploid *C. insueta* belongs to the genus *Cardamine*, which has long been studied for ecological polyploid speciation (Howard, 1948; Hussein, 1948), and represents adaptive radiation by recurrent polyploidization along water-usage gradients (Shimizu-Inatsugi et al., 2017; Akiyama et al., 2019). A major advantage to studying *Cardamine* is that it is closely related to the model plant *Arabidopsis thaliana*, and a reference genome assembly of *Cardamine hirsuta* (Gan et al., 2016) is publicly available, thus functional and genomic data of these model species are readily available. One allotriploid species in *Cardamine*, *C. insueta* (2*n* = 3*x* = 24; RRA), is a textbook example of contemporary speciation discovered by Urbanska-Worytkiewicz and Landolt (1974b). It was formed by the hybridization of two progenitor diploids *Cardamine amara* (2*n* = 2*x* = 16; AA) as a paternal progenitor and *Cardamine rivularis* (2*n* = 2*x* = 16; RR, belonging to *Cardamine pratensis* complex sensu lato) as a maternal progenitor approximately 100–150 years ago at the valley of Urnerboden in the Swiss Alps (Urbanska-Worytkiewicz and Landolt, 1972, 1974b; Urbanska et al., 1997; Mandáková et al., 2013; Zozomová-Lihová et al., 2014) (Figure 1A). The two diploid progenitors have distinct ecological habitats. While *C. amara* grows in and beside water streams, *C. rivularis* inhabits slightly moist sites, avoiding permeable and fast drying soil (Urbanska-Worytkiewicz and Landolt, 1974a,b) (Figure 1B). Around the end of the 19th to the early 20th centuries, the deforestation and land-use conversion to grazing induced the hybridization of these two diploids to produce the triploid species *C. insueta*, which is abundant in manured hay-meadows (Urbanska-Worytkiewicz and Landolt, 1972; Urbanska et al., 1997; Mandáková et al., 2013). Cytogenetic studies suggested that *C. insueta* served as a triploid bridge in the formation of pentaploid and hexaploid *Cardamine schulzii* by the further hybridization with autotetraploid *Cardamine pratensis* (*sensu stricto*, 2*n* = 2*x* = 30; PPPP; hypotetraploid derived from a chromosomal fusion) in Urnerboden (Mandáková et al., 2013).

The propagation of triploids mainly depends on vegetative propagation for two reasons, high male sterility *per se* and hay cutting and grazing in flowering season (Urbanska et al., 1997). One of the progenitor species, *C. rivularis*, can produce plantlets on the surface of leaves and nodes by ectopic meristem formation, which is a common feature of the *C. pratensis* complex (Smith, 1825; Salisbury, 1965; Dickinson, 1978). This characteristic is inherited by *C. insueta*, enabling it to be a dominant species at the site despite its ploidy level (Urbanska-Worytkiewicz and Landolt, 1974a; Urbanska et al., 1997). This type of leaf vivipary is only found in a limited number of angiosperms and assumed to contribute to population establishment in polyploids (Dickinson, 1978). In this sense, the trait of leaf vivipary can be considered a key factor for the establishment of this triploid.

Another interesting aspect of *C. insueta* establishment is its ecological niche shift relative to its progenitor species. Genus *Cardamine* is known to include many submergence tolerant species including *C. amara* (Shimizu-Inatsugi et al., 2017; Akiyama et al., 2019). An allotetraploid *Cardamine flexuosa*, derived from *C. amara* and *C. hirsuta* diploid progenitors, was shown to inherit parental traits and be successful in a wider soil moisture range (Shimizu-Inatsugi et al., 2017; Akiyama et al., 2019). The transcriptomic response of *C. flexuosa* to submergence or drought stress was shown to be combined although attenuated compared to its progenitor species, which could confer the wider tolerance found in the polyploid. Even though the niche separation between *C. rivularis* and *C. insueta* is not yet clearly illustrated, our field observations are consistent with this hypothesis.

In this study, we focused on the time-course gene expression pattern of the triploid *C. insueta* and its two diploid progenitors during submergence treatment, which induces both water stress and ectopic meristem formation on leaves. To study the time-course data of homeologs, we employed bioinformatic methods of variably expressed genes because data points of a time-course are not independent and serve partly as replicates (Yamaguchi et al., 2008; Shin et al., 2014). Here we combined the time course analysis with subgenome-classification bioinformatic workflow of HomeoRoq (Akama et al., 2014), and detected variably expressed homeologs (VEH) during the treatment. We address the following specific questions:

(1) What is the expression level and the ratio of homeologous genes in triploid species in response to submergence, either genome-wide or between each homeologous gene pair?
(2) Which kind of genes are enriched in VEH? Do they reflect the phenotypic trait of each progenitor species or the triploid? How does *C. insueta* combine the expression patterns of the two progenitors?

## Materials and Methods

### Plant Materials and RNA Sequencing

*Cardamine insueta*, *C. amara*, and *C. rivularis* plants used in this study were collected from Urnerboden. All plants were grown together in a plant cultivation room with 16 h light and 8 h dark cycle. The plants were planted in single pots, placed on trays, and watered from below.

Submergence treatment was started in the morning at 07:00. Two mature leaves were detached and submerged in water. We isolated RNA from the floating leaflets of the three species at nine time points after the start of submergence treatment (0, 2, 4, 8, 12, 24, 48, 72, and 96 h) using Qiagen RNeasy kit (Qiagen, Maryland, United States). RNA quality was assessed by Bioanalyser Nanochip (Agilent, Santa Clara, United States) and libraries quantified by Qubit (Thermo Fisher, Waltham, MA, United States). In total 27 libraries (3 species × 9 time points) were prepared according to NEBNext Ultra^*textTM*^ Directional RNA Library Prep Kit for Illumina (New England Biolabs, Ipswich, MA, United States) followed by paired end sequencing (100 bp × 2) on a HiSeq2000 with a HiSeq Paired-End Cluster Generation Kit and HiSeq Sequencing Kit (Illumina, San Diego, CA, United States). Trimmomatic (ver. 0.36) (Bolger et al., 2014) was used for discarding the low-quality reads with parameters of “PE -threads 4 -phred33 ILLUMINACLIP:adapters.fa:2:30:10 LEADING:20 TRAILING:20 SLIDINGWINDOW:4:20 MINLEN:50”.

### Reference Sequence Assembly

The reference sequences of *C. amara* genome (A-genome) and *C. rivularis* genome (R-genome) were assembled by single-nucleotide polymorphism (SNP) substitution at coding regions from the *C. hirsuta* genome (H-genome) (Gan et al., 2016) with the following steps. To assemble the reference sequence of A-genome, first, we pooled all RNA-Seq reads of the nine RNA-Seq samples of *C. amara*. Second, we mapped the reads onto the reference sequence (i.e., H-genome) using STAR (ver. 2.3.0e) (Dobin et al., 2013). Third, we detected SNPs and short indels from the mapping result using samtools (ver. 0.1.18) (Li et al., 2009). SNPs and indels were defined as the polymorphic loci where at least 80% of reads have the alternative nucleotides. Fourth, we replaced the nucleotides on the reference with the alternative nucleotides, if the alternative nucleotide was covered by at least five reads. Finally, the gene annotations of the assembled sequence were converted from the H-genome annotations with the replacement information. To improve the accuracy of sequence, we used the assembled sequence as a reference sequence, and repeated steps two through five, nine times. The resulting A-genome was used for the mapping of individual RNA-seq data from all three species. The R-genome was also reconstructed with the same protocol. As a result, 1,496,561 and 1,484,186 SNP regions on the H-genome were replaced for A-genome and R-genome, respectively.

### Evaluation of HomeoRoq Classification Confidence Using Diploids

We used HomeoRoq (ver. 2.1) (Akama et al., 2014) to classify genomic origins of homeolog-specific reads in the nine *C. amara* and *C. rivularis* samples. Following the HomeoRoq pipeline, for each *C. amara* sample, we used STAR to map reads onto the A-genome and R-genome and used HomeoRoq to classify reads as *A-origin*, *R-origin*, and *unclassified*. Then, we calculated the percentage of misclassified reads (i.e., the reads that were classified as *R-origin*). Similarly, we used HomeoRoq to calculate the percentage of misclassified reads (i.e., the reads that were classified as *A-origin*) in each *C. rivularis* sample.

### Homeolog Expression Quantification and A-Origin Ratio Definition of Triploid

We used HomeoRoq to analyze the nine *C. insueta* samples. For each *C. insueta* sample, we used STAR to map reads onto A-genome and R-genome and used HomeoRoq to classify reads as *A-origin*, *R-origin*, and *unclassified*. Then, we customized HTSeq (Planet et al., 2012) to count the number of read pairs that mapped on homeolog region for *A-origin*, *R-origin*, and *unclassified* reads of each *C. insueta* sample separately. In the customized HTSeq, if a read mapped on the region overlapped by multiple homeologs, a read was divided by the number of homeologs.

To calculate the number of fragments per kilobase mapped (FPKM) for *C. insueta* samples (*I*_A_ and *I*_R_ samples), we first allocated the *unclassified* reads into *A-origin* and *R-origin* reads with A-origin ratio. A-origin ratio of homeolog *h* at the time point *S* was defined as $p_{\text{h}}^{\text{s}}=a_{\text{h}}^{\text{s}}/\left(a_{\text{h}}^{\text{s}}+r_{\text{h}}^{\text{s}}\right)$, where $a_{\text{h}}^{\text{s}}$ and $r_{\text{h}}^{\text{s}}$ are the numbers of *A-origin* and *R-origin* reads of homeolog *h* at the time point *S*, respectively. Thus, the number of *A-origin* reads after *unclassified* reads allocation ($a_{\text{h}}^{\prime ,\text{s}}$) was calculated as $a_{\text{h}}^{\prime ,\text{s}}=a_{\text{h}}^{\text{s}}+u_{\text{h}}^{\text{s}}\,p_{\text{h}}^{\text{s}}$, where $u_{\text{h}}^{\text{s}}$ is the number of *unclassified* reads of homeolog *h* in sample *S*. Similarly, $r_{\text{h}}^{\prime ,\text{s}}=a_{\text{h}}^{\text{s}}+u_{\text{h}}^{\text{s}}\,\left(1-p_{\text{h}}^{\text{s}}\right)$ for *R-origin* reads. Then, FPKM of *A-origin* reads of homeolog *h* in sample *S* was calculated as $10^{9}\,a_{\text{h}}^{\prime ,\text{s}}/\left(L_{\text{h}}^{\text{A}}\,A^{\text{s}}\right)$, where $L_{\text{h}}^{\text{A}}$ is the length of homeolog *h* on A-genome and *A*^s^ is the total number of *A-origin* reads in sample *S*; likewise, FPKM of *R-origin* reads was calculated as $10^{9}\,a_{\text{h}}^{\prime ,\text{s}}/\left(L_{\text{h}}^{\text{R}}\,R^{\text{s}}\right)$, where $L_{\text{h}}^{\text{R}}$ is the length of homeolog *h* on R-genome and *R*^s^ is the total number of *R-origin* reads in sample *S*.

In addition, FPKM of progenitors were calculated from the total number of reads (i.e., $a_{\text{h}}^{\text{s}}+u_{\text{h}}^{\text{s}}+r_{\text{h}}^{\text{s}})$. Therefore, FPKM of *C. amara* and *C. rivularis* were calculated as $10^{9}\,\left(a_{\text{h}}^{\text{s}}+u_{\text{h}}^{\text{s}}+s_{\text{h}}^{\text{s}}\right)/\left(L_{\text{h}}^{\text{A}}\,A^{\text{s}}\right)$ and $10^{9}\,\left(a_{\text{h}}^{\text{s}}+u_{\text{h}}^{\text{s}}+s_{\text{h}}^{\text{s}}\right)/\left(L_{\text{h}}^{\text{A}}\,R^{\text{s}}\right)$, respectively.

### Expressed Homeologs and PCA Analysis

An expressed homeolog was defined as a homeolog with FPKM > 1.0. A homeolog expressed in a sample [i.e., either *amara*-derived in *C. insueta* (*I*_A_), *rivularis*-derived in *C. rivularis* (*I*_R_), *C. amara* or *C. rivularis*] was defined as a homeolog with FPKM > 1.0 at least at one of the nine time points. In total, 21,131 homeologs were expressed at least in one sample. PCA was performed against log_10_-transformed FPKM of these 21,131 expressed homeologs. To avoid calculating log_10_0, the log_10_-transfromed FPKM was truly calculated as log_10_(FPKM + 1).

### Identification of Variably Expressed Homeologs (VEH) and Gene Ontology (GO) Enrichment Analysis

Mean and coefficient of variation (CV) were calculated from log_10_-transformed FPKM over the nine time points. VHE was defined as an homeolog satisfied the mean > 1.0 and the CV > 0.20. We identified from *I*_A_, *I*_R_, *C. amara*, and *C. rivularis* samples, separately.

Gene ontology (GO) enrichment analysis was performed for the four variably expressed homeolog (VEH) sets with R packages clusterProfiler (ver. 3.12.0) and org.At.tair. db (ver. 3.8.2) (Yu et al., 2012). To remove redundancies of GO categories, only GO categories which are associated with 10–500 *Cardamine* homeologs and below the third level in the GO category hierarchy were used. The threshold FDR = 0.1 was used for cutoff of significantly enriched GO categories.

## Results

### Plantlet Induction on *C. insueta* and *C. rivularis* Leaves by Submergence

At the field of Urnerboden valley, we could scarcely observe normal seed setting on *C. insueta*, but small plantlets on leaves were frequently observed after flowering, as described previously (Urbanska, 1977). We also observed small plantlets on the leaves of *C. rivularis*. In contrast, *C. amara* does not form plantlets on leaves, rather adventitious roots and shoots were formed from rhizomes. In the natural habitat, the plantlet formation of *C. rivularis* and *C. insueta* can be seen at flowering to post-flowering season (Salisbury, 1965; Urbanska, 1977). It was also reported that *C. pratensis* (which is closely related to *C. insueta* or considered the same species) tend to bear more plantlets on the leaves in damper sites than in drier sites (Salisbury, 1965), implying that high moisture could be the trigger for meristem formation. Thus, we tested plantlet induction by submergence treatment using dissected leaves with this trio of species in the lab. We detached mature leaves from mother plants propagated in a climate chamber and floated the leaves on water. Within 16 h, we observed the activation of dormant shoot meristems and initiation of ectopic root meristems, which formed visible plantlets on *C. rivularis* leaves 96 h after submergence (Supplementary Figure S1 and Supplementary Dataset S1). Induction of ectopic plantlets followed a similar time-course in *C. insueta*. In contrast, plantlet induction was not observed on the leaves of *C. amara*. In addition, during the 96-h treatment, no symptoms of necrosis appeared on any of the leaves, suggesting that all three species have some submergence tolerance for at least 96 h.

### Gene Annotation on the Two Diploid Progenitor Reference Sequences

To detect how homeologous genes are expressed in plantlet induction and submergence treatment, we harvested time-course RNA-Seq samples of *C. insueta* and diploid progenitor leaves at nine time points after initial submergence (i.e., 0, 2, 4, 8, 12, 24, 48, 72, and 96 h) (Supplementary Figure S2A). We harvested the first lateral leaflet pair in young leaves with no ectopic plantlets. To quantify homeolog-specific gene expression, we assembled the genomes of *C. amara* (A-genome) and *C. rivularis* (R-genome), respectively, using the same pipeline of a reference-guided approach using RNA-Seq reads (Supplementary Figure S2B). The genome sequence of a close relative, *C. hirsuta* (H-genome) (Gan et al., 2016), was used as a reference. The A-genome structure is reported to be almost perfectly collinear with that of H-genome, except for one pericentric inversion at chromosome 1, by cytological studies (Mandáková et al., 2013, 2014). The genome structures of the A-genome and R-genome are also similar to each other (Mandáková et al., 2013). The length of assembled reference sequences of A-genome and R-genome are 198,651,635 and 198,654,862 nucleotides, respectively, which are nearly the same as the length of the original H-genome (198,654,690 nucleotides). We also annotated the orthologous genes of *C. amara* and *C. rivularis* according to the information of *C. hirsuta* H-genome. In total, we found 23,995 and 24,115 genes covered by at least one read among the nine time points on the assembled A-genome and R-genome, respectively. These gene sets, which correspond to 81.5 and 81.7% of 29,458 genes in H-genome, respectively, were defined as expressed and used for the following analysis.

### Expression Ratio From Each Subgenome Is Consistent With the Number of Chromosomes

We applied the HomeoRoq analysis pipeline (Akama et al., 2014) to map RNA-Seq reads of *C. insueta* samples to A-genome and R-genome, and classify the origin of each RNA-seq read of *C. insueta* samples to either *A-origin* (i.e., the genomic origin of the read is A-subgenome) or *R-origin* (Supplementary Figure S2C). After filtering for read quality, 10.6 million read pairs on average among the nine samples could be classified as homeolog-specific read pairs (Supplementary Dataset S2). Of the total homeolog-specific read pairs in the *C. insueta* 0 h sample, 27.3 and 56.7% of read pairs were classified as *A-origin* and *R-origin*, respectively. To confirm that *A-origin* and *R-origin* reads were correctly mapped to A-genome and R-genome, respectively, we checked the alignments of several highly expressed homeologs in the mapping results with Integrative Genomics Viewer (IGV) (Supplementary Figure S3) (Robinson et al., 2011). We found only a few SNPs in the alignments between *A-origin* reads and A-genome, and *R-origin* reads and R-genome, respectively. Considering the young origin of *C. insueta* within 150 years, we can assume that the genomic distances between *C. insueta* and its progenitors are very small, and HomeoRoq can manage this range of difference with low error rate (Kuo et al., 2020). Besides *A-origin* and *R-origin* reads, the remaining 16.0% of read pairs could be classified to neither *A-origin* nor *R-origin* (*unclassified*) due to the lack of SNPs or the identical sequence on the correspondence region. As a whole genome, the ratio of *A-origin* to *R-origin* reads was approximately 1:2.

When we analyzed all samples from the other eight time points, we observed a slight increase in the proportion of *A*-origin reads in correlation with the time point, from 1:2.07 at 0 h to 1:1.90 at 96 h (Supplementary Dataset S2). Instead of this minor transition, the expression ratio between subgenomes remained A:R ≈ 1:2 with *C. insueta* samples at all time points, indicating that the expression ratio from each subgenome is consistent with the number of chromosome regardless of the submergence treatment.

### Most Homeolog Pairs Were Expressed in Proportion to the Subgenomes in *C. insueta*

**FIGURE 2:**
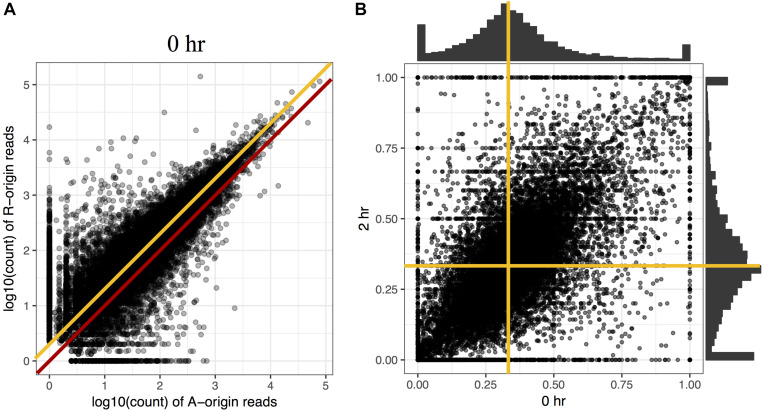
Comparison of homeolog expression from A- and R-subgenomes. **(A)** Expression ratio between A- and R-homeologs before submergence treatment in the triploid *C. insueta*. Each dot shows the relation between the log10-transformed A-origin and R-origin read of a homeolog pair at 0 h point. Only the homeolog pairs with FPKM > 1.0 in either *I*_A_ or *I*_R_ samples are shown. The red line represents the ratio *A*:*R* = 1:1, and the orange line represents the ratio *A*:*R* = 1:2. **(B)** Comparison of A-origin ratios between two time points, 0 and 2 h, in the triploid *C. insueta*. Each point shows the A-origin ratios of a homeolog pair at 0 and 2 h. The orange lines represent the position of *A*-origin = 0.33 at each time point.

To investigate the proportion of expression levels of homeolog pairs in *C. insueta*, we quantified the expression level of each homeolog pair at each time point. We found that (i) the correlation between the expression levels of homeolog pairs was higher than 0.81 at any time point (Figure 2A and Supplementary Figure S4). However, (ii) the expression levels of most homeologs expressed from the A-subgenome (A-homeolog) were approximately half that of R-homeologs. To understand the proportion of expression levels of homeolog pairs in detail, we calculated A-origin ratio—the proportion of A-homeolog expression level to the total A-homeolog and R-homeolog expression levels—for all homeolog pairs at each time point. We found that the distribution of A-origin ratios had a gentle peak at the position of 0.33 at all time points (Figure 2B and Supplementary Figure S5). This result suggests the expression ratio of the majority of homeolog pairs is consistent with the copy number, i.e., the subgenome-set numbers of the triploid. In addition, we found two sharp peaks at both edges, the positions of 0.0 and 1.0, of A-origin ratio, which represent the homeologs only expressed in either subgenome.

Additionally, to investigate whether the A-origin ratio changes during the submergence treatment, we compared the A-origin ratio distributions between different time points. The patterns of all time points were correlated to each other, with the least coefficiency (0.66) between 0 and 2 h (Figure 2B and Supplementary Table S1). This result indicates that A-origin ratios did not change drastically in most homeolog pairs by the submergence treatment, but a limited number of homeolog pairs change the expression balance.

### The Whole Genome Expression Pattern of Each *C. insueta* Subgenome Is Closer to That of Its Progenitor Genome

**FIGURE 3:**
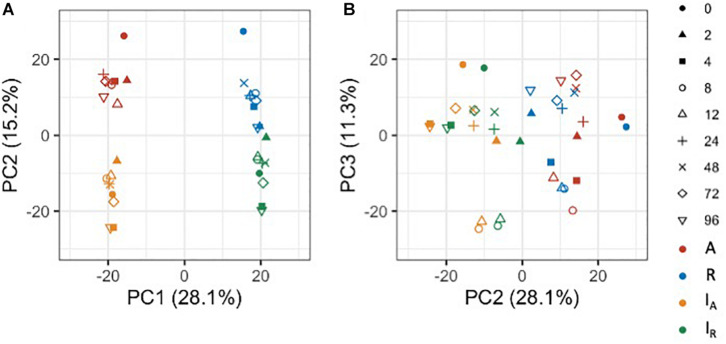
Principal component analysis of the expressed homeologs/genes in *C. insueta* (*I*_A_), *C. insueta* (*I*_R_), *C. amara* (A) and *C. rivularis* (R) samples at 9 time points. PCA was performed against log_10_-transformed FPKM of 21,131 expressed homeologs. The two plots show the relation between PC1-PC2 **(A)** and PC2-PC3 **(B)**. The colors represent genome/subgenome, and the shapes represent the time points after the start of submergence treatment.

To gain an overview of how homeologous gene expression varies at the whole genome level among *C. insueta* and the progenitor species *C. amara* and *C. rivularis*, we conducted principal component analysis (PCA). PCA was performed against the log_10_-transformed FPKM of 21,131 expressed homeologs (Figure 3). We found that the first principal component (PC1) grouped samples into two groups: the one with A-homeologs of *C. insueta* (*I*_A_) and *C. amara* (*A*) samples and the other with R-homeologs of *C. insueta* (*I*_R_) and *C. rivularis* (*R*) samples. In addition, we also found that the second principal component (PC2) grouped samples into two groups: one consisting of polyploid samples (*I*_A_ and *I*_R_ samples, lower side of Figure 3A) and the other consisting of diploid samples (*A* and *R* samples, upper side of Figure 3A). By PC1 and PC2, the samples were grouped into four clusters according to the subgenome type. In contrast, by PC2 and the third principal component (PC3), we observed the transition according to the treatment time, showing a characteristic transition from 0 to 12 h, and the recurrence of 24, 48, 72, and 96 h samples toward 0 h samples in each subgenome, which might reflect the combined effect of submergence stress and circadian rhythm (Figure 3B). The result of PC1 suggests that the majority of the homeologs of *I*_A_ and *I*_R_ should retain a similar expression pattern to each parent, *A* and *R*. When we focus on PC2, the distance between *R* and *I*_R_ is slightly closer than that between *A* and *I*_A_. This might reflect the difference in the number of subgenome sets in the triploid, *A*:*R* = 1:2, implying a stronger effect from the progenitor with more subgenome sets.

### VEHs Related to Submergence and Their GO Enrichment Analysis

To understand the difference among species in plantlet formation on the leaf and in submergence response, we focused on the homeologs with a higher expression change during the treatment. Standard tools to identify differentially expressed genes between different conditions are not directly applicable to time-course data, in which expression levels of neighboring time points may be highly correlated. We defined variably expressed homeologs (VEHs) according to the coefficient of variation (CV) among the expression levels of the nine time points, since CV is used for identifying variably expressed genes in various studies involving time-course analysis (Czechowski et al., 2005; Yamaguchi et al., 2008; Shin et al., 2014; Zhao et al., 2017). We identified 1,194, 1,144, 1,030, and 1,063 VEHs from *I*_A_, *I*_R_, *A*, and *R* genome/subgenome with the cutoff CV > 0.2 throughout the treatment, respectively (Supplementary Dataset S3). We visualized the patterns by focusing on two genes that were expected to be affected (Supplementary Figure S6). The genes associated with ethylene-response such as *ERF1* (AT3G23240) (Chao et al., 1997; Solano et al., 1998) and circadian rhythm such as *CCA1* (AT2G46830) (Alabadí et al., 2001) were identified as VEHs in all samples, which should reflect the ethylene-response to submergence and circadian rhythm response, respectively. The expression pattern of these two homeologs were similar among all four VEH sets from *I*_*A*_, *I*_*R*_, *A*, and *R* (Supplementary Figure S6). In addition to these common VEHs, we also found more homeologs identified as VEHs only in one to three samples (Supplementary Figure S7).

To investigate the biological processes of VEH sets of *I*_*A*_, *I*_*R*_, *A*, and *R*, we performed gene ontology (GO) enrichment analysis against the four VEH sets (Table 1 and Supplementary Dataset S4). The numbers of enriched GO categories were 146, 155, 160, and 181, respectively for *I*_*A*_, *I*_*R*_, *A*, and *R*. A value of negative log10(*q*-value) more than 1 was defined as significant, and a higher value indicates stronger enrichment. We found that some of the GO categories related to water stress, including GO:0006066 (alcohol metabolic process), GO:0009723 (response to ethylene) and GO:0009414 (response to water deprivation), were enriched in all four VEH sets (Table 1). As gas diffusion rates are restricted under water, submergence of plants induces ethylene accumulation and low oxygen availability, which could result in the reorganization of the ethylene-response pathway and fermentation pathway (e.g., anaerobic respiration and alcohol metabolism). The enriched categories GO:0006066 and GO:0009723 indicate that *I*_*A*_, *I*_*R*_, *A*, and *R* all respond to ethylene and hypoxia signals with the submergence treatment. Two alcohol related categories (GO:0006066 alcohol metabolic process and GO:0046165 alcohol biosynthetic process) were more strongly enriched in *A* and *I*_*A*_, which was two orders of magnitude higher than *I*_R_ and *R* [>2 difference in negative log10(*q*-value) in Table 1]. In addition, GO:0009414 (response to water deprivation), which encompasses the expression changes of aquaporin genes and ethylene-responsive genes (Supplementary Dataset S5), was enriched.

**TABLE 1:** The negative log10(*q*-value) of the enriched GO categories in each VEH set described in the manuscript.

**Keyword**	**Accession**	**VEH set**				**GO name**
		***A***	***I*_*A*_**	***I*_*R*_**	***R***	
Alcohol	GO:0006066	6.9	5.2	2.7	2.0	Alcohol metabolic process
	GO:0046165	6.4	6.4	4.4	2.7	Alcohol biosynthetic process
Water	GO:0009414	3.9	3.3	5.1	7.3	Response to water deprivation
Ethylene	GO:0009723	7.9	3.6	2.8	7.5	Response to ethylene
	GO:0009873	6.1	15	ND	2.5	Ethylene-activated signaling pathway
	GO:0071369	6.1	1.3	ND	2.3	Cellular response to ethylene stimulus
	GO:0009692	2.6	ND	ND	ND	Ethylene metabolic process
	GO:0009693	2.6	ND	ND	ND	Ethylene biosynthetic process
Abscisic acid	GO:0009738	1.4	ND	ND	ND	Abscisic acid-activated signaling pathway
	GO:0071215	1.4	ND	ND	ND	Cellular response to abscisic acid stimulus
Oxidative stress	GO:0006979	19	ND	ND	2.6	Response to oxidative stress
	GO:0042743	ND	ND	ND	3.0	Hydrogen peroxide metabolic process
	GO:2000377	ND	1.6	1.5	5.6	Regulation of reactive oxygen species metabolic process
	GO:0010310	ND	1.3	1.1	5.1	Regulation of hydrogen peroxide metabolic process
Meristem	GO:0035266	ND	3.1	1.9	ND	Meristem growth
	GO:0010075	ND	3.4	2.0	ND	Regulation of meristem growth
	GO:0048509	ND	2.8	1.8	ND	Regulation of meristem development

*This data is the extract from the list of all enriched GOs (Supplementary Dataset S4). ND means that the category was not detected as enriched by the threshold FDR = 0.1.*

In contrast, some GO categories related to submergence stress were only above the significance threshold in part of the four VEH sets with various combinations (Table 1). The two categories related to ethylene metabolism, GO:0009873 (ethylene-activated signaling pathway) and GO:0071369 (cellular response to ethylene stimulus), were not detected in *I*_*R*_ but all other three. All these ethylene related GO categories were most strongly enriched in *A*, suggesting larger number of genes are detected than other VEH sets. In addition, the categories related to abscisic acid signaling, which is known to work antagonistically to ethylene, GO:0009738 (abscisic acid-activated signaling pathway) and GO:0071215 (cellular response to abscisic acid stimulus), were also detected only in *A* with many inactivated genes by treatment. In contrast, the categories related to oxidative stress showed the strongest enrichment in *R* than others, suggesting higher intensity of oxidative stress in *C. rivularis* than other species.

### VEHs Related to Meristem and Their GO Enrichment Analysis

Among the GO categories enriched in four VEH sets, three categories were related to meristem activity: GO:0035266 (meristem growth), GO:0010075 (regulation of meristem growth), and GO:0048509 (meristem development) (Table 1). They were enriched only in VEH sets of *I*_A_ and *I*_R_, but not in *A* and *R*, although *C. rivularis* can also produce ectopic meristems.

**FIGURE 4:**
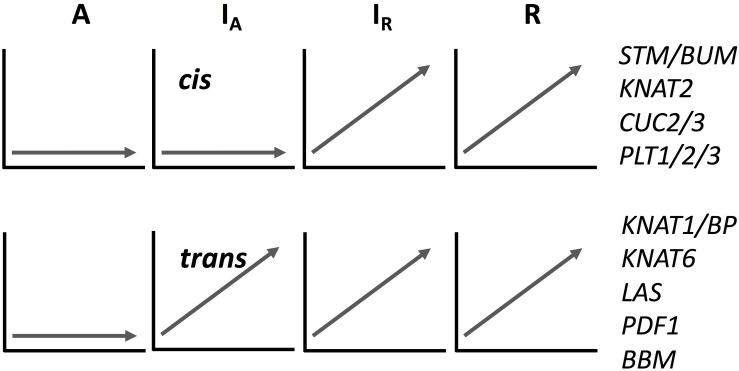
A schematic drawing of *cis-* and *trans-*regulation of key regulatory genes in meristem formation. The expression patterns of *I*_A_ homeologs are arbitrary categorized to *cis-* or *trans-*regulated according to the expression pattern of each homeolog. See Supplementary Figure S8 for their original temporal expression patterns.

We analyzed the expression pattern of several known transcriptional factors which could be involved in ectopic meristem formation and development in *Cardamine* (Figure 4 and Supplementary Figure S8). Class I Knotted1-like homeobox (KNOX) transcription factors function to maintain shoot apical meristem activity in many different plant species (Vollbrecht et al., 1991; Long et al., 1996; Hay and Tsiantis, 2010). Importantly, the overexpression of *SHOOTMERISTEMLESS* (*STM*) and another KNOX gene, *Arabidopsis knotted 1*-like gene (*KNAT1*), are known to cause ectopic meristem formation on the leaf in *A*. *thaliana* (Chuck et al., 1996; Williams, 1998). Moreover, an *STM* ortholog is required for leaf vivipary in *Kalanchoë daigremontiana* (Garcês et al., 2007), a clonal propagation trait that is also observed in *C. rivularis* and *C. insueta*. As summarized in Figure 4, orthologs of the four *A. thaliana* KNOXI genes, *STM*, *KNAT1*, *KNAT2 and KNAT6*, showed upregulated expression in all or some of *I*_A_, *I*_R_ and *R*, but not in *A*. In addition, we also found that *PDF1* increased expression levels in *C. insueta* (both *I*_A_ and *I*_R_) and *R*, which is exclusively detected in the L1 layer of shoot apical meristem throughout the shoot development of *Arabidopsis* (Abe et al., 1999). Three other transcription factor-encoding genes, *CUC2*, *CUC3*, and *LAS*, which contribute to ectopic shoot apical meristem in tomato leaves (Rossmann et al., 2015), were induced in *R* and *I*_R_ but not in *A*.

The expression of genes related to root apical meristem maintenance and formation showed similar patterns to those related to shoot apical meristem formation. Transcription factors with an AP2/ERF domain for the maintenance of root apical meristem [*PLT1*, *PLT2*, and *PLT3*; (Drisch and Stahl, 2015)], were scarcely expressed in *A* but induced in others. For genes responsible for radial patterning, *SHR* and *SCR*, expression level of *SHR* was increased in all four sets, while that of *SCR* was upregulated only temporarily and reverted in 24 h. The expression of *WOX5*, a key factor to maintain the root stem cell (Sarkar et al., 2007), was very low in all sets, most probably due to the extremely limited expression area only at the quiescent center.

Many of the above-mentioned transcription factors contributing to meristem formation and maintenance are known to be related to or controlled by auxin, thus the transportation of auxin might be also involved in ectopic meristem development in *C. insueta* and *C. rivularis*. One of the auxin transporter genes, *PIN1*, was induced by the treatment in all four sets soon after the start of submergence, but after 24 h the high expression level was only retained in *I*_A_, *I*_R_, and *R*. On the other hand, the other auxin transporter genes *PIN3*, *PIN4*, and *PIN7* were temporarily induced by the treatment, but soon decreased among all sets. The peaks of expression of these three genes were at 4–12 h in *A*, 12–24 h in *R*, and 8–24 h in *I*_A_ and *I*_R_, suggesting involvement in meristem formation and development. By the VEH enrichment analysis, GO:0060918 (auxin transport) and GO:0009926 (auxin polar transport) were enriched in *I*_A_ and *I*_R_. In contrast, the other two auxin related GO categories, GO:0009850 (auxin metabolic process) and GO:0009851 (auxin biosynthetic process), were enriched in only two parents, *A* and *R*.

## Discussion

### Applicability of HomeoRoq to Diverse Ploidy Levels

HomeoRoq was developed to classify genomic origins of RNA-Seq reads of allopolyploids consisting of two subgenomes (Akama et al., 2014), and has already been applied to *Arabidopsis kamchatica* (2*n* = 4*x* = 32; HHLL), an allotetraploid between two diploids of *Arabidopsis halleri* (2*n* = 2*x* = 16; HH) and *Arabidopsis lyrata* (2*n* = 2*x* = 16; LL). Here, we successfully applied HomeoRoq to another species with a different ploidy level. The average proportions of the reads mapped on the wrong genome in *C. amara* and *C. rivularis* samples were 1.1 ± 0.1% and 1.2 ± 0.1%, respectively (Supplementary Dataset S2). This high accuracy is comparable to the evaluation of the *A. kamchatica* data, 1.23–1.64% (Akama et al., 2014; Kuo et al., 2020).

The proportion of *unclassified* reads in this study, which has the same matching rates on both parental genomes, was very close to that in the *A. kamchatica* study. In this study, 11.5 ± 2.0% of reads in *C. insueta* samples were *unclassified* on average, compared to 11.0% in *A. kamchatica* (Akama et al., 2014), suggesting a similar divergence level between subgenomes in the two cases. Considering the percentage of unclassified reads and the low misclassification rate with diploid progenitors, HomeoRoq can be applied to genomes of any ploidy level providing that the genome consists of two types of subgenome.

### Total Gene Expression Level of Each Subgenome Is Consistent With the Chromosome Number

The ratio of *A-origin* to *R-origin* reads in *C. insueta* was approximately 1:2. This result is consistent with the distribution of A-origin ratio showing a gentle peak at around 0.33 with a smooth decrease toward the edges (Figure 2). This distribution indicates that expression ratios of most homeologs correlates with the copy number. A similar tendency could be found in other Brassicaceae allotetraploids (Akama et al., 2014; Douglas et al., 2015; Yang et al., 2016). In the analysis of triploid banana (2*n* = 3*x* = 33; ABB), a hybrid between *Musa acuminata* (2*n* = 2*x* = 22; AA) and *Musa balbisiana* (2*n* = 2*x* = 22; BB), the read proportion is distributed around 0.66 for the B alleles by 155 homeologs with rather high expression level detected by LC-MSMS as isoforms (van Wesemael et al., 2018). This could also be seen in hexaploid bread wheat consisting of three subgenomes, where 70% of genes showed balanced expression among homeologs (Ramírez-González et al., 2018). So far, this consistency between ploidy number and expression ratio looks like a general rule in many species with some exceptions like tetraploid cotton (Yoo et al., 2013).

In addition to the majority of genes that show balanced expression between homeologs, a limited proportion of genes show significant differential expression. Even though a direct comparison among studies is difficult due to different thresholding policies, the number of genes with unbalanced homeolog expression tends to be the minor fraction in many quantitative studies. Further studies should show whether a similar pattern is observed in even higher ploidy levels or other odd ploidies.

### Limited Number of Homeolog Pairs Changed Expression Ratio in Submergence Condition

Though the number of homeologs with unbalanced expression is smaller than that with balanced expression, they could play a significant role in speciation of polyploid species, especially for achieving a combined trait from progenitors. A series of studies have reported that homeolog expression ratios can be changed depending on external environments (Bardil et al., 2011; Dong and Adams, 2011; Akama et al., 2014; Paape et al., 2016). Akama et al. (2014) evaluated the changes of the homeolog expression ratio of *A. kamchatica* after cold treatment. They reported that the homeolog expression ratios before and after cold treatment were highly correlated (*R*^2^ = 0.87), and only 1.11% of homeolog pairs statistically significantly changed in expression ratios in response to cold treatment (Akama et al., 2014). A similar result was reported for zinc treatment of *A. kamchatica.* The correlation of homeolog expression ratios between zinc treatment and control ranged from 0.89 to 0.94, and 0.3–1.5% of homeologs significantly changed expression ratios after Zn treatment (Paape et al., 2016).

In this study using another Brassicaceae species, *C. insueta*, the correlation coefficients of A-origin ratios between 0 h and the other time-points ranged from 0.68 to 0.82 (Supplementary Table S1). The lowest correlation occurring between 2 h and other time points may suggest that the initial reaction to the treatment had the strongest effect on gene expression. The overall high correlations among time points indicate that the expression ratios of most homeologs do not change considerably in response to treatment. Even though *C. rivularis* and *C. amara* show species-specific responses to submergence, leaf vivipary and submergence tolerance, respectively, no specific expression preference or dominance of either progenitor was detected in the triploid. This suggests that transcriptional changes in only a limited number of homeologs, rather than genome-wide, might be responsible for the control of physiological change under submergence conditions.

### Triploid Inherited Advantageous Traits From Progenitors

Only about 6% of the expressed genes were detected as VEH throughout the 96-hr treatment in each genome and subgenome, suggesting the criteria were fairly conservative. Among enriched GO categories are water stress related ones, particularly ethylene-response and fermentation. Fermentation metabolism in plants is important for submergence stress. We found more VEH genes in the fermentation-related categories in the diploid *C. amara* and the *amara*-derived subgenome of *C. insueta* than counterparts (Table 1). This suggests that *C. insueta* inherited the fermentation ability as a submergence response more largely from *C. amara* side. The ethylene signaling pathway should have been stimulated in all three species as many related GO categories are found enriched in all VEH genes. However, the stress level seems to be variable according to the species as shown in the difference of enriched GO categories. In all of these ethylene related GO categories, *C. amara* had the strongest enrichment (i.e., highest number of VEH genes), and the enrichment in *amara*-derived subgenome was stronger than in *rivularis*-derived subgenome in *C. insueta*. These enrichment intensities should suggest that *C. amara* has higher acclimation ability to submergence through an activation of alcohol metabolic pathway and alteration in hormone signaling pathway and thus suffer from less oxidative stress as a result, as speculated by its habitat and a previous study (Shimizu-Inatsugi et al., 2017). In addition, in *C. insueta*, the contribution to the stress response of *I*_A_ seems larger than that of *I*_R_, found as stronger enrichment in *I*_A_ than in *I*_R_.

GO enrichment analysis with VEH genes also showed three GO categories related to meristem, GO:0035266 (meristem growth), GO:0010075 (regulation of meristem growth) and GO:0048509 (meristem development). They were only enriched in the VEH sets of *I*_A_ and *I*_R_, but not above the significance threshold in two parents, despite the fact that *C. rivularis* also produces plantlets on the leaf by the activation of ectopic meristems. This might imply that the ability to form ectopic plantlets in response to submergence is enhanced in the triploid *C. insueta* compared to the diploid *C. rivularis*. Considering the disadvantage in sexual reproduction due to the odd ploidy, effective vegetative propagation through plantlets might have been critically important for *C. insueta*.

The expression pattern of known key regulatory genes that function to maintain meristem activity showed two typical patterns, as shown in Figure 4 and Supplementary Figure S8, although mixed patterns are also found. Expression of both types of genes was upregulated in *C. rivularis* (*R*, Figure 4) but not in *C. amara* (*A*, Figure 4) in response to submergence. Expression of these genes was also upregulated in the *C. insueta* subgenome *I*_R_, but followed two different patterns in the *I*_A_ subgenome. These patterns could be categorized as either non-induced, similar to *C. amara*, or induced, similar to *C. rivularis*, suggesting that non-induced homeologs could be *cis-*regulated (i.e., difference in the *cis*-regulatory regions derived from the two progenitors), and induced homeologs could be *trans-*regulated by *I*_R_. One possibility is that this difference reflects the developmental timing of gene expression during meristem formation and the divergence of *cis*-regulation. For example, the *cis-*regulated genes *STM* and *CUC2* are expressed earlier during embryogenesis in *A. thaliana* than the *trans*-regulated genes *KNAT6* and *KNAT1/BP* (Hay and Tsiantis, 2010). This variation might imply a regulatory relationship among these genes in the gene regulatory network controlling plantlet formation in *C. insueta* leaves. This type of information might provide insights that warrant further study into the molecular mechanism of leaf vivipary in *C. rivularis* and *C. insueta*.

## Data Availability Statement

The datasets generated for this study can be found in DNA Data Bank of Japan (DDBJ) Sequence Read Archive (DRA), www.ddbj.nig.ac.jp [accession no. DRA009830].

## Author Contributions

JiS analyzed the data in consultation with KS. JiS and RS-I wrote the manuscript. RS-I, AH, KKS, and JuS refined the manuscript. HH performed the experiments. AH, KKS, and JuS supervised the project. All authors read, corrected, and approved the manuscript.

## Conflict of Interest

JuS is the CEO of the company Humanome Lab. The remaining authors declare that the research was conducted in the absence of any commercial or financial relationships that could be construed as a potential conflict of interest.

## Supplementary Material

Supplementary Figure 1 Leaf vivipary in a representative leaf of *C. rivularis.* Anew plantlet was first visible 96 hours after submergence (circled, shown in close-up below). Plantlets initiated from dormant shoot meristems (shoot meristem with visible leaf shown). Shoot growth was detected 8 hours after submergence, followed by root initiation (16 hours). The shoot and root poles appeared to fuse (72 hours), followed by shoot and root growth to produce a plantlet (96 hours).

Supplementary Figure 2 Overview of homeolog expression analysis. **(A)** To monitor homeolog expression profiles during submergence responses, we sequenced 27 RNA samples extracted from the leaf of the three species (*C. insueta*, *C. amara*, and *C. rivularis*) at the nine time points after the start of submergence. **(B)** A-genome and R-genome were assembled from RNA-Seq reads of the nine *C. amara* and *C. rivularis* samples, respectively. **(C)** Homeolog-specific expression was quantified using HomeoRoq pipeline. For each *C. insueta* sample, reads were mapped onto both A-genome and R-genome. Then, homeolog-specific reads were classified into *A-origin*, *R-origin* and unclassified (with the same mismatch rates on A and R) according to the number of mismatches on the two mapping results. After classification, the read count data, FPKM (fragments per kilobase of exon per million reads mapped), and A-origin ratio were calculated from the results.

Supplementary Figure 3 Examples of mapping alignments. Alignments of RNA-seq read mapping of the four homeologs *ETR1*, *PIN3*, *CCA1*, and *PDF1* at 0 hr are visualized with Integrative Genomics Viewer (IGV). The top panel of each subfigure shows the alignments of *C. amara* reads and *A-origin* reads of *C. insueta* mapped to A-genome. The bottom panel shows the alignments of *C. rivularis* reads and *R-origin* reads of *C. insueta* mapped to R-genome.

Supplementary Figure 4 Expression ratio between A- and R-homeologs during submergence treatment in the triploid *C. insueta*. Each dot shows the relation between the log_10_-transformed A-origin and R-origin reads of a homeolog pair at nine time points. Only the homeolog pairs with FPKM > 1.0 in either *I*_*A*_ or *I*_*R*_ samples are shown. The orange line represents the ratio A:R=1:2.

Supplementary Figure 5 Distributions of A-origin ratio of *C. insueta* at nine time points. The width of the bin is 0.025 in these histograms. The vertical orange lines indicate one-third of A-origin ratios. The number of homeologs for plotting histograms is shown in the title of each histogram.

Supplementary Figure 6 Time-course changes of homeolog expression. Expression profiles of *ERF1* and *CCA1*. Top panels represent expression of the four homeologs in the I_*A*_, I_*R*_, *C. amara*, and *C. rivularis* samples.

Supplementary Figure 7 Overlaps of the number of VEH genes/homeologs of four genomes/subgenomes.

Supplementary Figure 8 Time-course changes of homeolog expression of plantlet associated homeologs. Expression profiles of known meristem associated homeologs are shown in line charts. Red line indicates that the gene/homeolog was identified as VEH.

Supplementary Table 1 Pearson correlation coefficients of A-origin ratios. Pearson correlation coefficients of A-origin ratios between two *C. insueta* samples among the nine time points.

Supplementary Dataset 1 Videos for visualizing of plantlet initiation of *C. insueta* Video file (MP4) visualizes the plantlet initiation of *C. insueta* at the incubator under the standard condition with the 16 h light and 8 h dark. Leaflet were detached form *C. insueta* individuals and floated on the water in a beaker. Photos were taken between May 16, 2018 and June 07, 2018, with 90 min intervals. Photos taken at daylight were concatenated into video.

Supplementary Dataset 2 Statistics of RNA-Seq data processing An Excel format file that contains the statistics of RNA-Seq data processing with HomeoRoq pipeline. Sheet 1: Number of read pairs before and after quality controls with Trimmomatic; Sheet 2: Number of reads that were mapped onto A-genome and R-genome, and number of reads that were classified into A-origin, R-origin, and unclassified reads with HomeoRoq.

Supplementary Dataset 3 FPKM and CV of variably expressed homeologs an Excel format file that contains gene names, averages of log10-transformed FPKM, and coefficient of variation (CV) of log10-transformed FPKM of variably expressed homeologs (VEHs). Sheet 1: VEHs of IA samples; Sheet 2: VEHs of IR samples; Sheet 3: VEHs of *C. amara* samples; Sheet 4: VEHs of *C. rivularis* samples.

Supplementary Dataset 4 Enriched GO terms of variably expressed homeologs an Excel format file that contains gene ontology (GO) enrichment analysis results of VEHs. Sheet 1: Summarization of GO enrichment analysis results. Values in cells represent negative log10(*q*-value); Sheet IA: GO enrichment analysis result of VEHs of *I*_A_ samples; Sheet IR: GO enrichment analysis result of VEHs of *I*_R_ samples; Sheet A: GO enrichment analysis result of VEHs of *C. amara* samples; Sheet R: GO enrichment analysis result of VEHs of *C. rivularis* samples.

Supplementary Dataset 5 Relative gene expression levels of the genes of the category GO:0009414 (response to water deprivation).
