# Supplementary material for: A Recently Formed Triploid Cardamine insueta Inherits Leaf Vivipary and Submergence Tolerance Traits of Parents
Source: Front Genet. 2020 Oct 6;11:567262. doi: 10.3389/fgene.2020.567262 (PMC7573311; doi:10.3389/fgene.2020.567262)

Supplementary Material

**Supplementary Table S1**

**Pearson correlation coefficients of A-origin ratios.** Pearson correlation coefficients of A-origin ratios between two *C. insueta* samples among the nine time points.

|  | 2 hr | | 4 hr | | 8 hr | | 12 hr | | 24 hr | | 48 hr | | 72 hr | | 96 hr | |
| --- | --- | --- | --- | --- | --- | --- | --- | --- | --- | --- | --- | --- | --- | --- | --- | --- |
| 0 hr | 0.663 | | 0.807 | | 0.689 | | 0.707 | | 0.757 | | 0.791 | | 0.794 | | 0.732 | |
| 2 hr |  | | 0.685 | | 0.672 | | 0.693 | | 0.716 | | 0.722 | | 0.697 | | 0.639 | |
| 4 hr |  | |  | | 0.745 | | 0.762 | | 0.778 | | 0.800 | | 0.811 | | 0.747 | |
| 8 hr |  | |  | |  | | 0.833 | | 0.759 | | 0.773 | | 0.749 | | 0.695 | |
| 12 hr |  | |  | |  | |  | | 0.787 | | 0.798 | | 0.771 | | 0.719 | |
| 24 hr |  | |  | |  | |  | |  | | 0.839 | | 0.820 | | 0.735 | |
| 48 hr |  | |  | |  | |  | |  | |  | | 0.839 | | 0.760 | |
| 72 hr |  | |  | |  | |  | |  | |  | |  | | 0.805 | |
|  | |  | |  | |  | |  | |  | |  | |  | |  |

**Supplementary Figure S1**

**Leaf vivipary in a representative leaf of *C. rivularis.*** A new plantlet was first visible 96 hours after submergence (circled, shown in close-up below). Plantlets initiated from dormant shoot meristems (shoot meristem with visible leaf shown). Shoot growth was detected 8 hours after submergence, followed by root initiation (16 hours). The shoot and root poles appeared to fuse (72 hours), followed by shoot and root growth to produce a plantlet (96 hours).


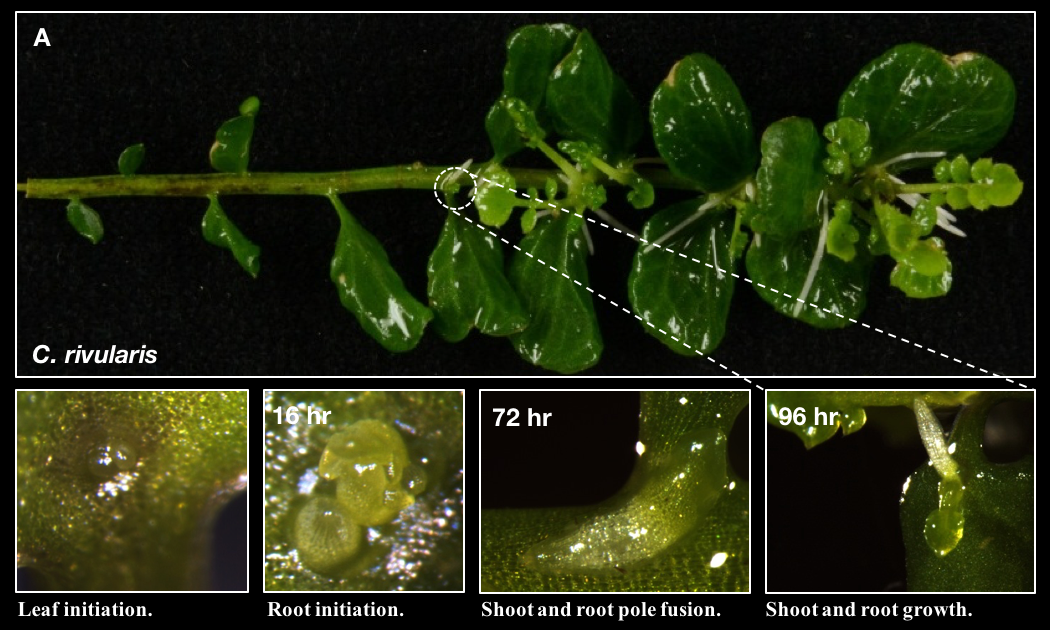


**Supplementary Figure S2**

**Overview of homeolog expression analysis.** (***A***) To monitor homeolog expression profiles during submergence responses, we sequenced 27 RNA samples extracted from the leaf of the three species (*C. insueta*, *C. amara*, and *C. rivularis*) at the nine time points after the start of submergence. (***B***) A-genome and R-genome were assembled from RNA-Seq reads of the nine *C. amara* and *C. rivularis* samples, respectively. (***C***) Homeolog-specific expression was quantified using HomeoRoq pipeline. For each *C. insueta* sample, reads were mapped onto both A-genome and R-genome. Then, homeolog-specific reads were classified into *A-origin*, *R-origin* and unclassified (with the same mismatch rates on A and R) according to the number of mismatches on the two mapping results. After classification, the read count data, FPKM (fragments per kilobase of exon per million reads mapped), and A-origin ratio were calculated from the results.

**Supplementary Figure S3**

**Examples of mapping alignments.** Alignments of RNA-seq read mapping of the four homeologs *ETR1*, *PIN3*, *CCA1*, and *PDF1* at 0hr are visualized with Integrative Genomics Viewer (IGV). The top panel of each subfigure shows the alignments of *C. amara* reads and *A-origin* reads of *C. insueta* mapped to A-genome. The bottom panel shows the alignments of *C. rivularis* reads and *R-origin* reads of *C. insueta* mapped to R-genome.

**
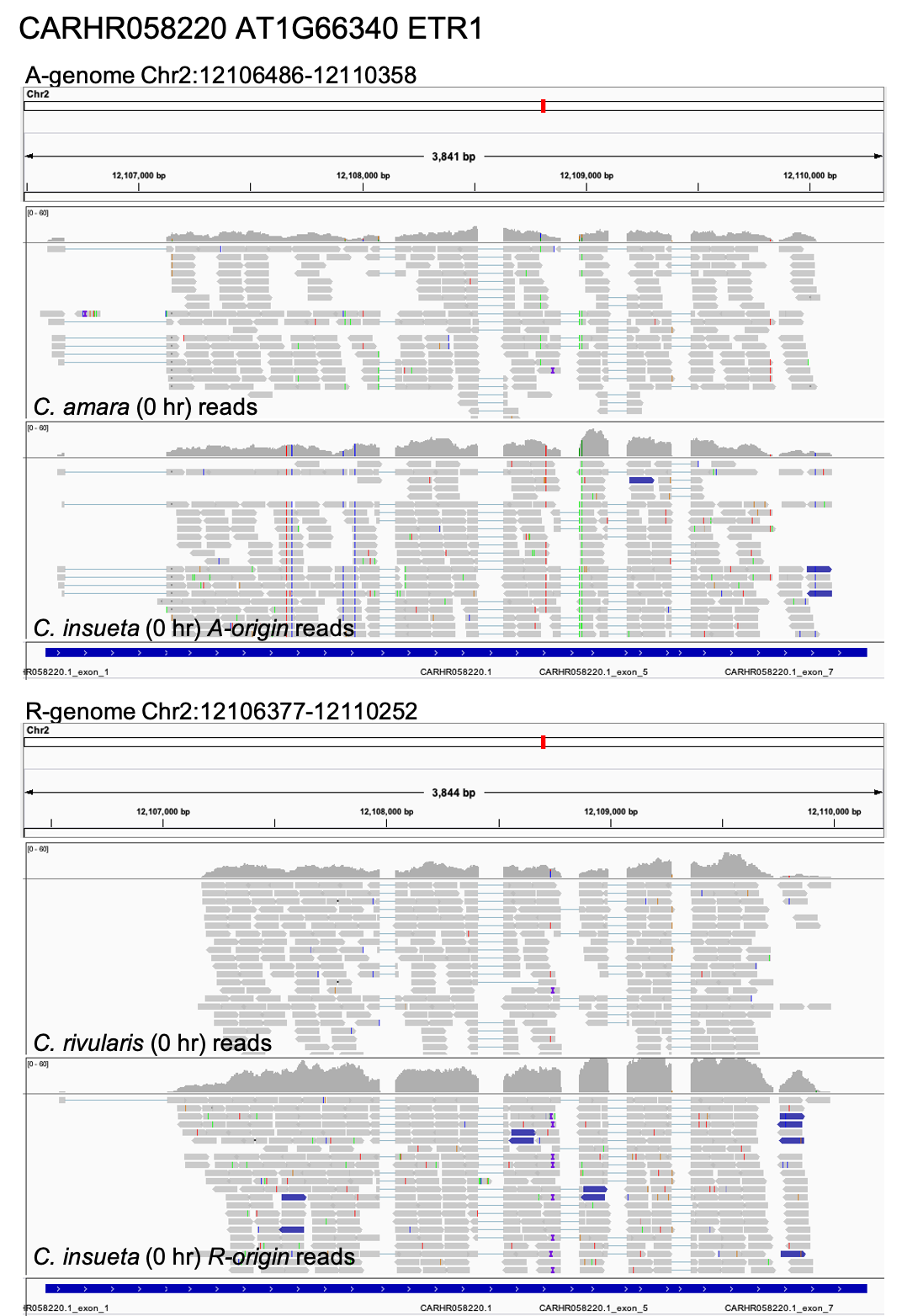
**

**
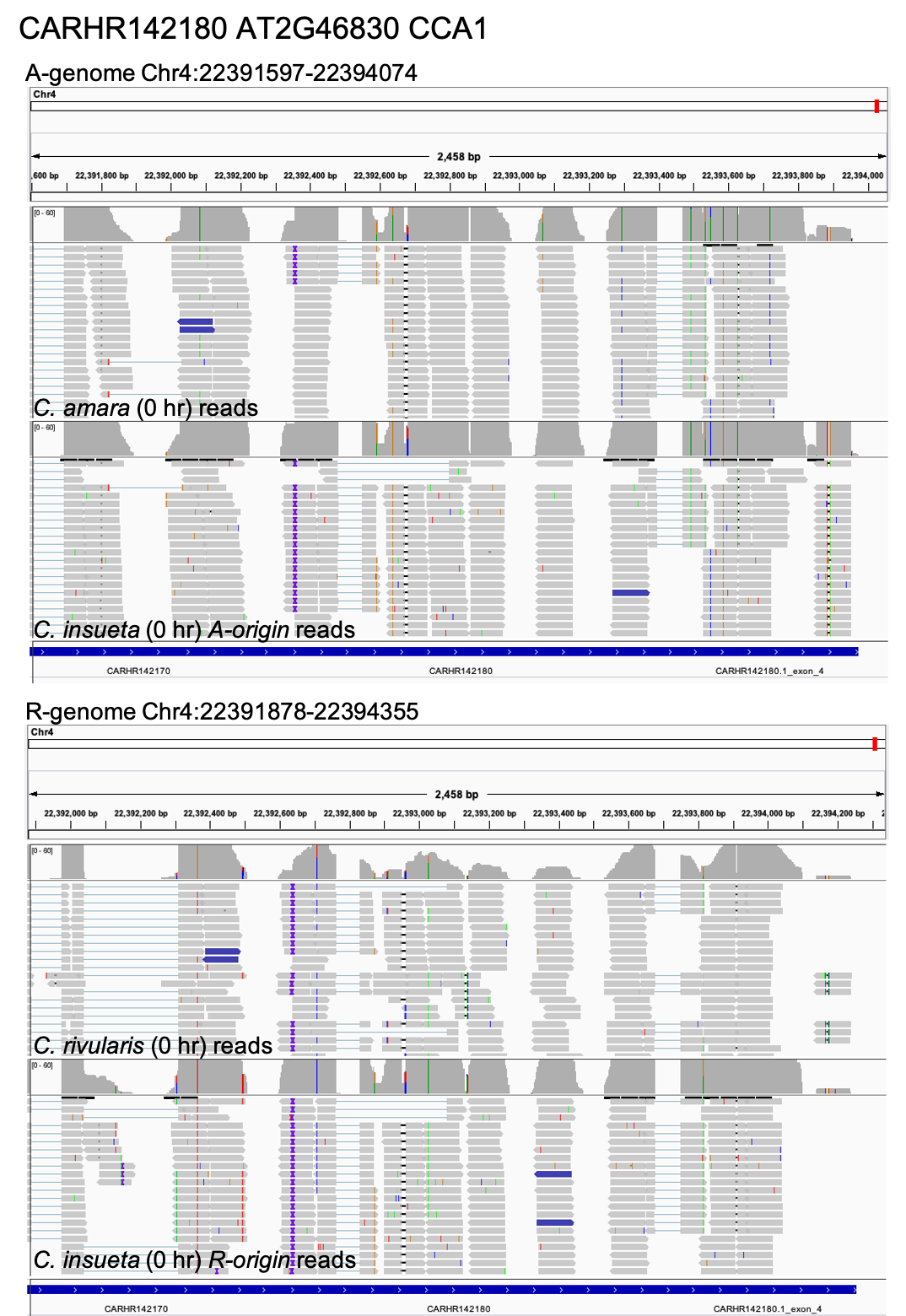
**

**
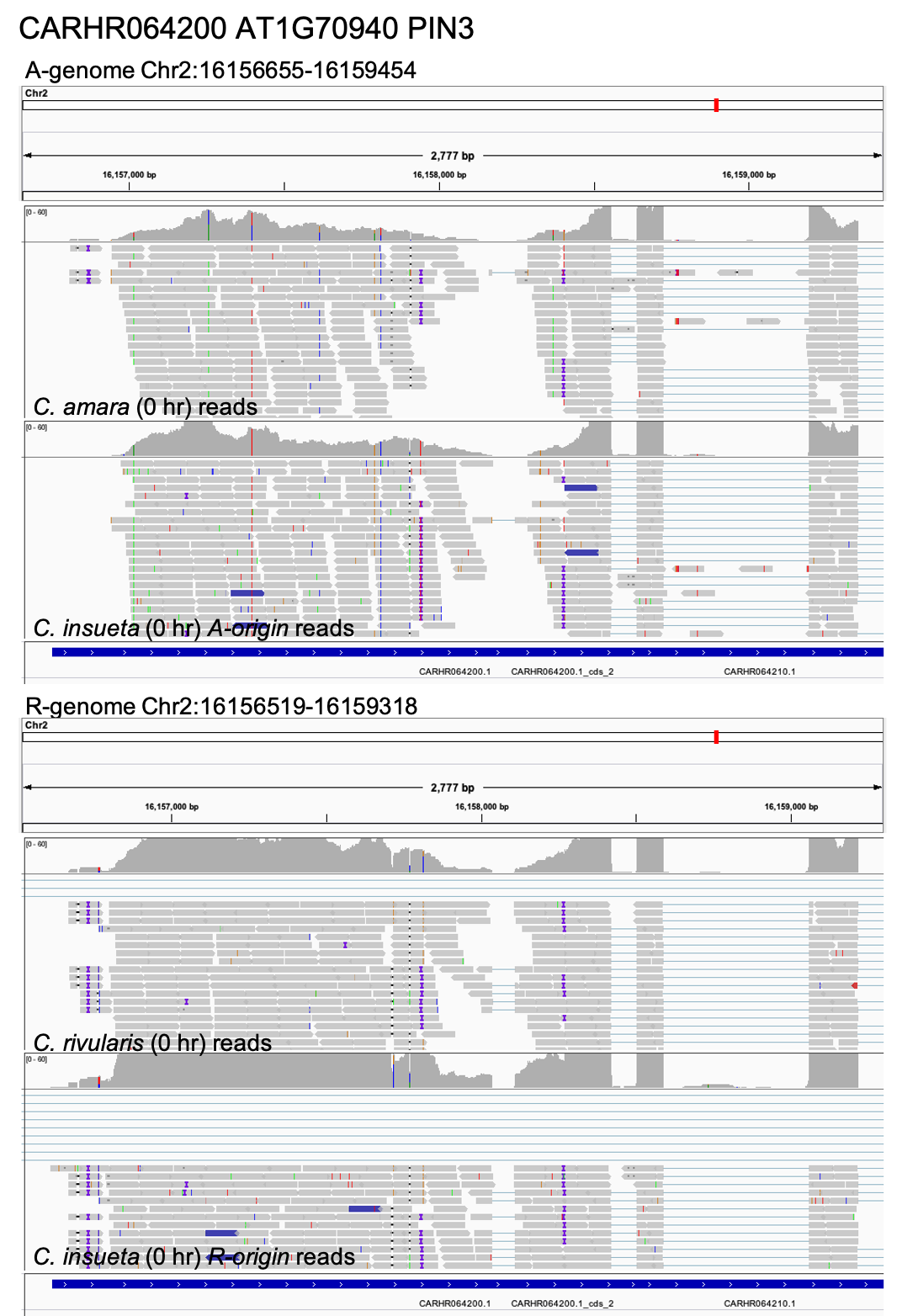
**

**
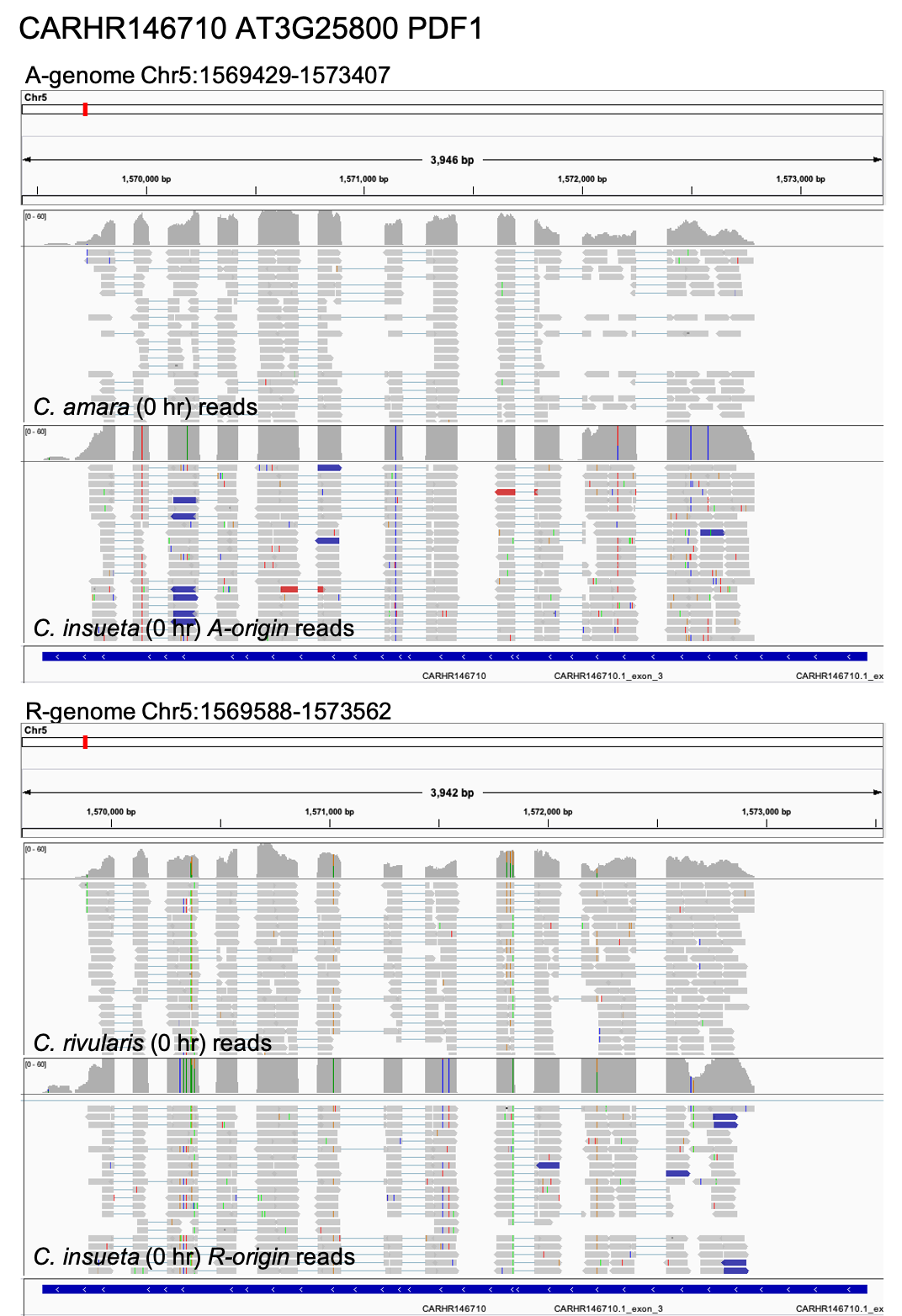
**

**Supplementary Figure S4**

**Expression ratio between A- and R-homeologs during submergence treatment in the triploid *C. insueta*.** Each dot shows the relation between the log_10_-transformed A-origin and R-origin reads of a homeolog pair at nine time points. Only the homeolog pairs with FPKM > 1.0 in either *I_A_* or *I_R_* samples are shown. The orange line represents the ratio A:R=1:2.


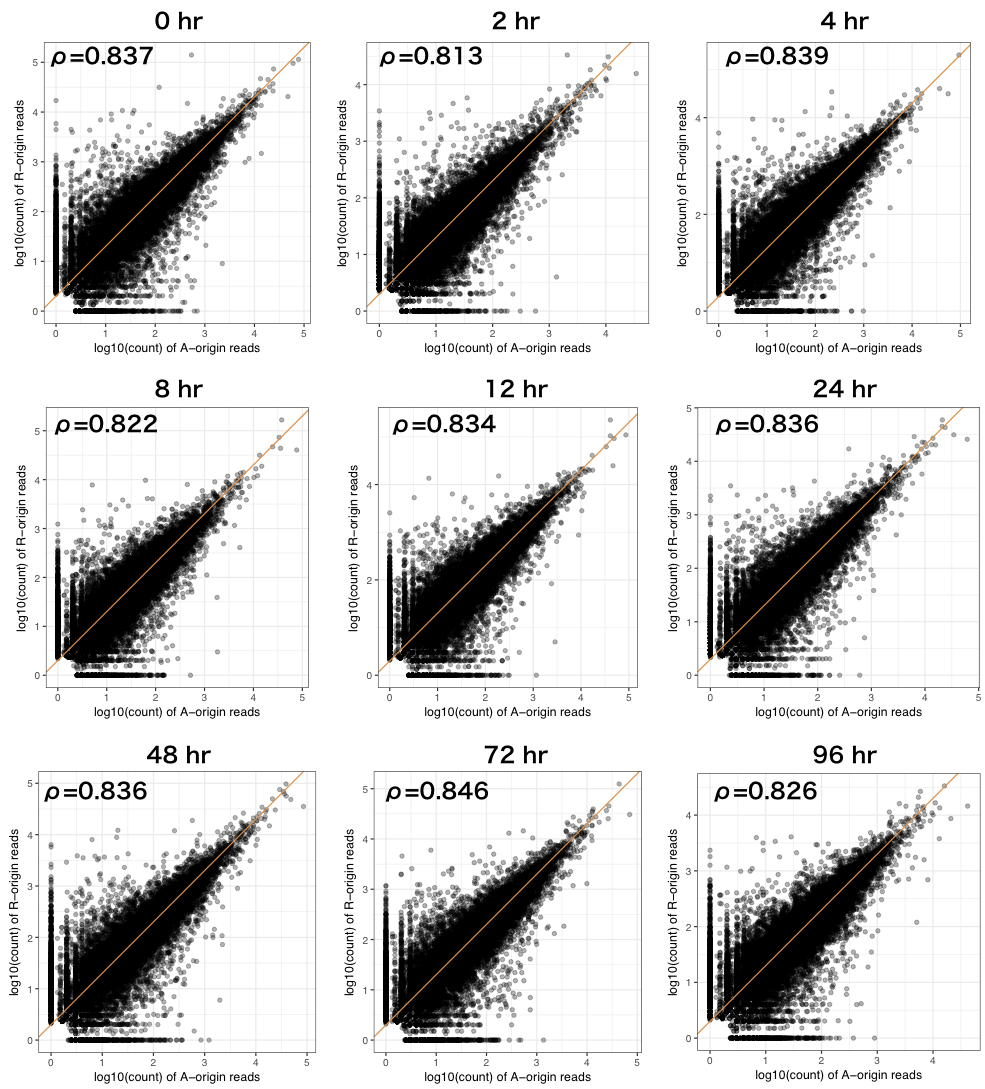


**Supplementary Figure S5**

**Distributions of A-origin ratio of *C. insueta* at nine time points.** The width of the bin is 0.025 in these histograms. The vertical orange lines indicate one-third of A-origin ratios. The number of homeologs for plotting histograms is shown in the title of each histogram.

**Supplementary Figure S6**

**Time-course changes of homeolog expression.** Expression profiles of *ERF1* and *CCA1*. Top panels represent expression of the four homeologs in the I_A_, I_R_, *C. amara*, and *C. rivularis* samples.


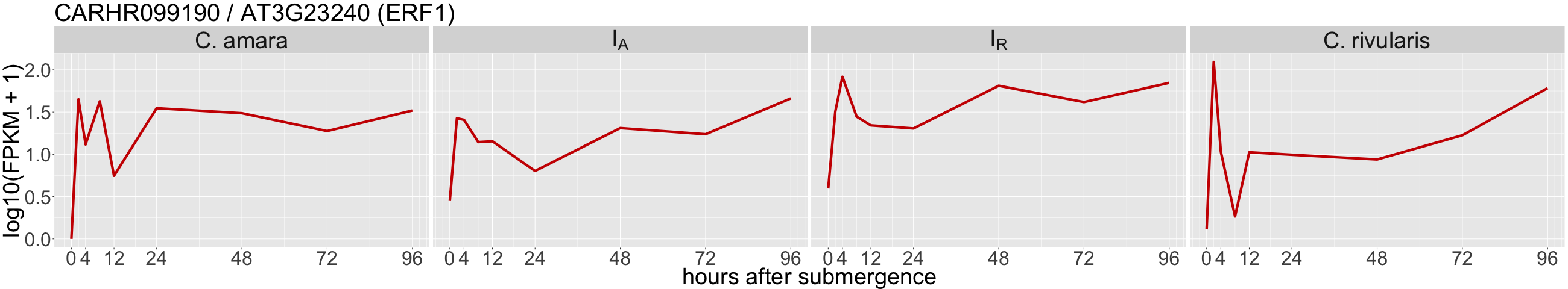


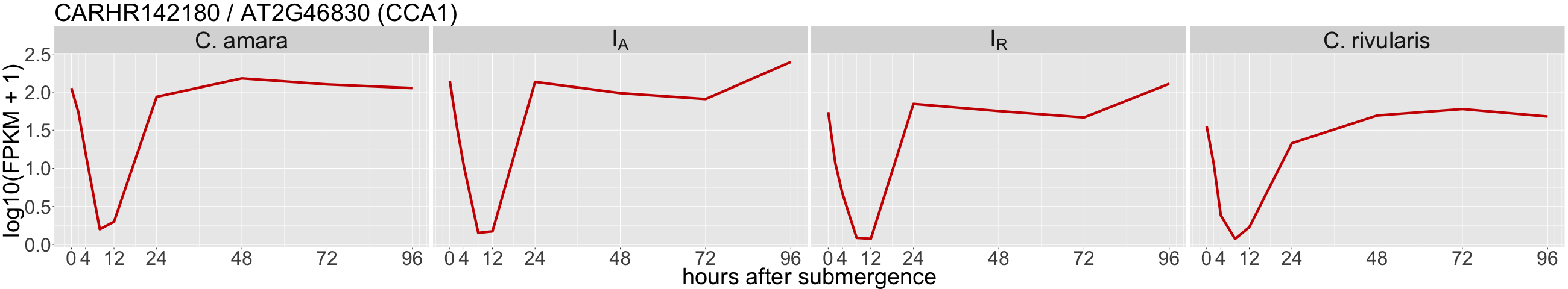


**Supplementary Figure S7**

**Overlaps of the number of VEH genes/homeologs of four genomes/subgenomes.**


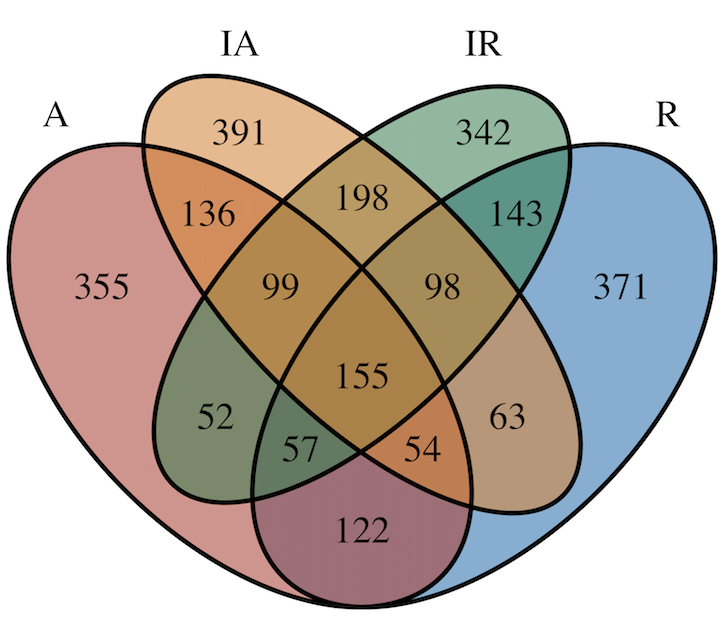


**Supplementary Figure S8**

**Time-course changes of homeolog expression of plantlet associated homeologs.** Expression profiles of known meristem associated homeologs are shown in line charts. Red line indicates that the gene/homeolog was identified as VEH.

The following genes were not included in this figure because they are not annotated in *Cardamine* genomes: *ANT, REV*, *CORONA*, *PIN2*, *WUS* and *CLV3*. These genes were also not included because the expression were zero in all samples: WOX10/CARHR021160 and WOX11/CARHR077710.


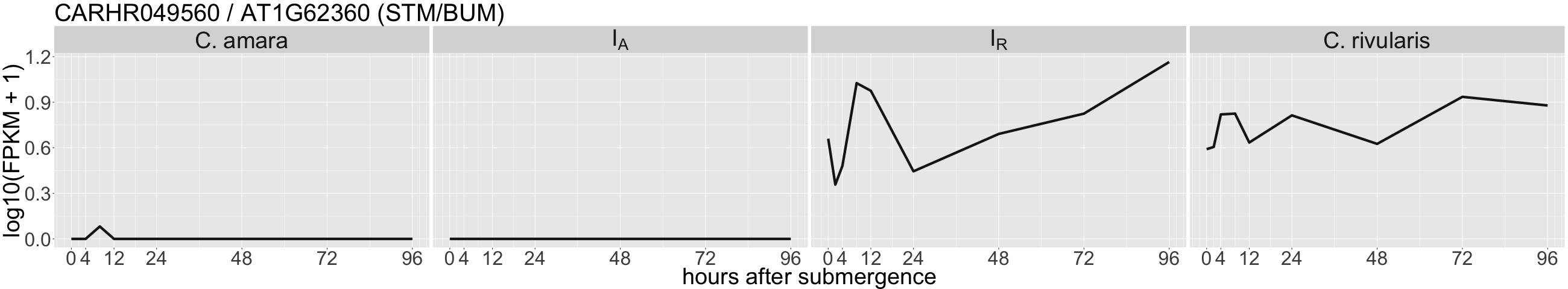


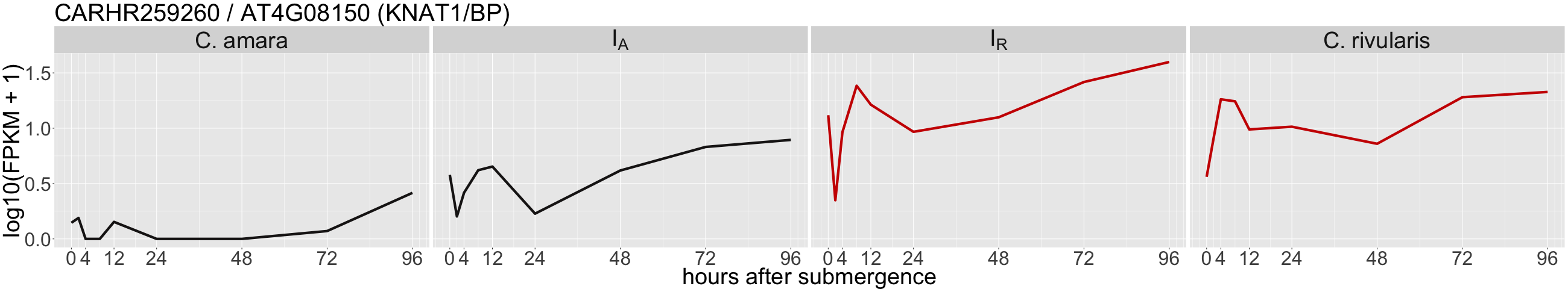

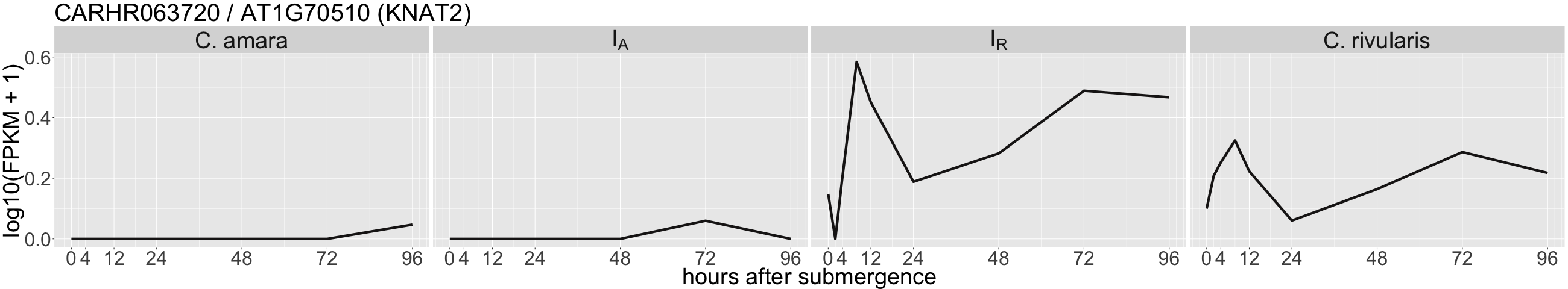

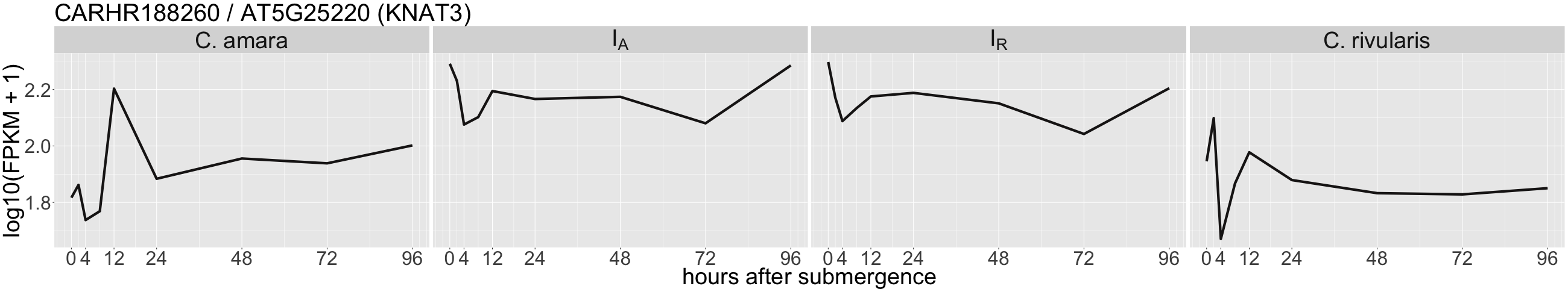

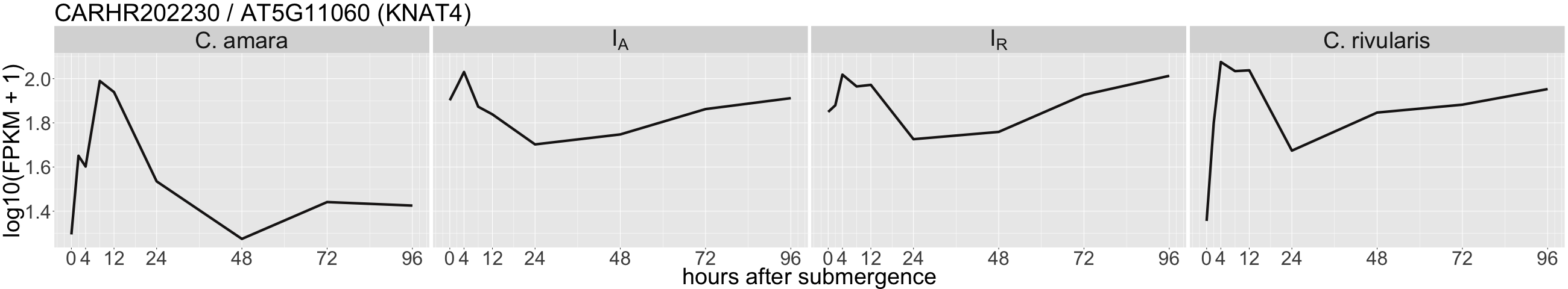

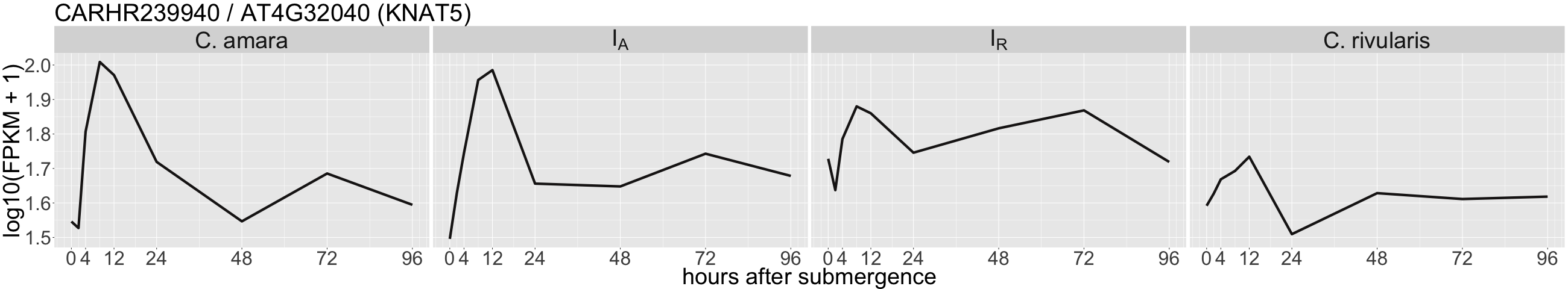

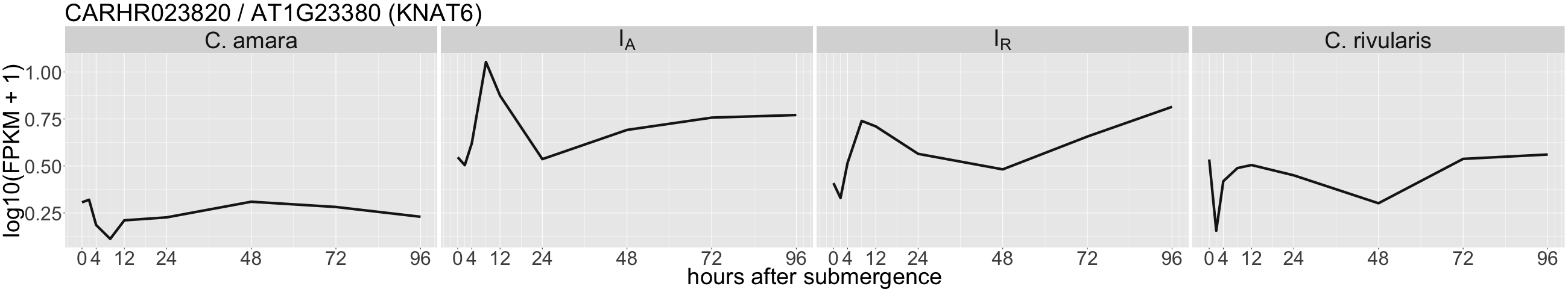

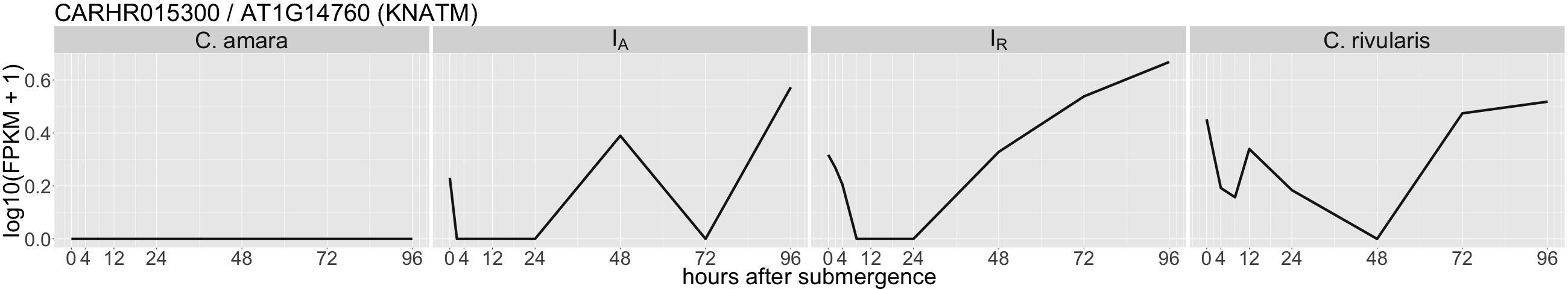

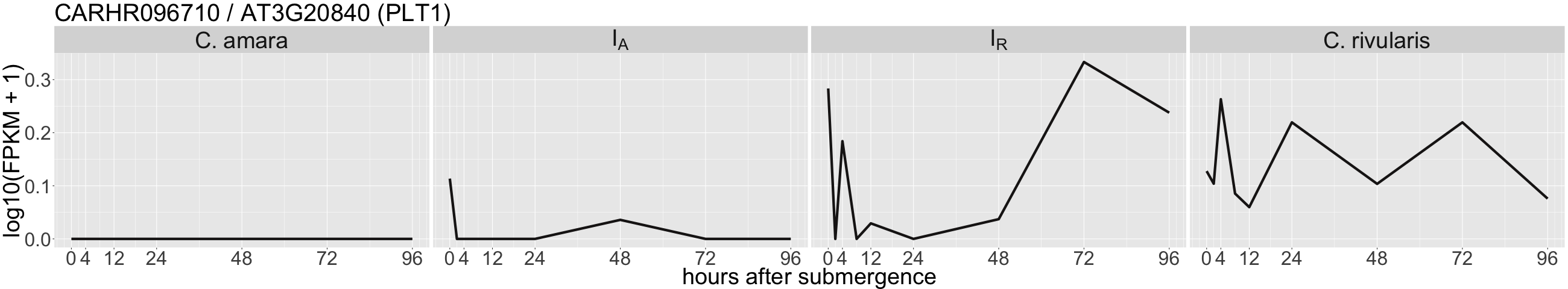

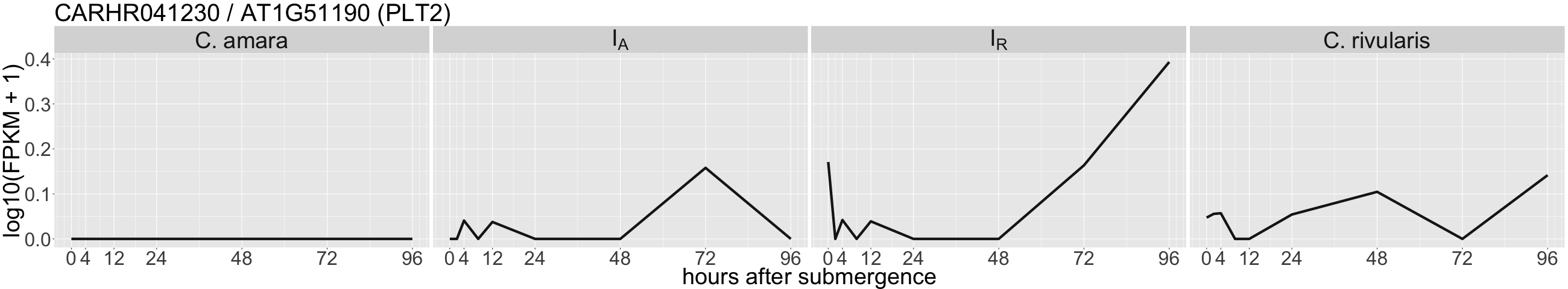

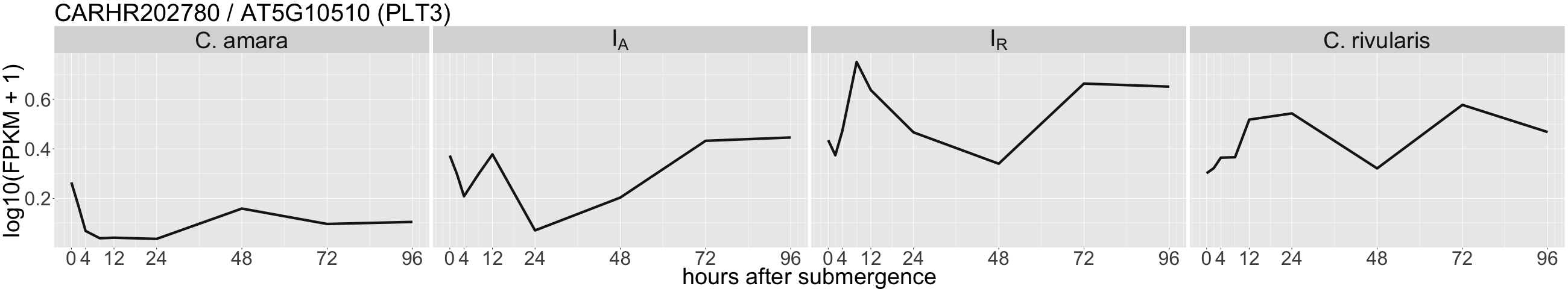

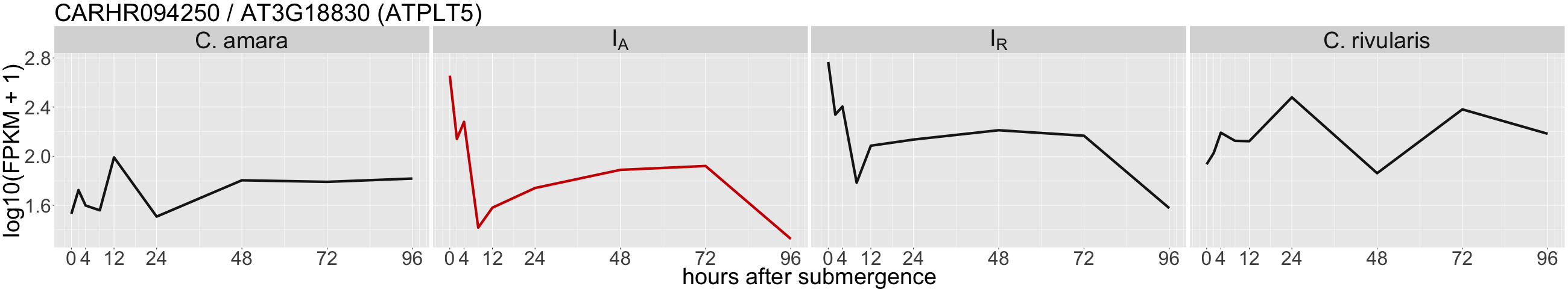

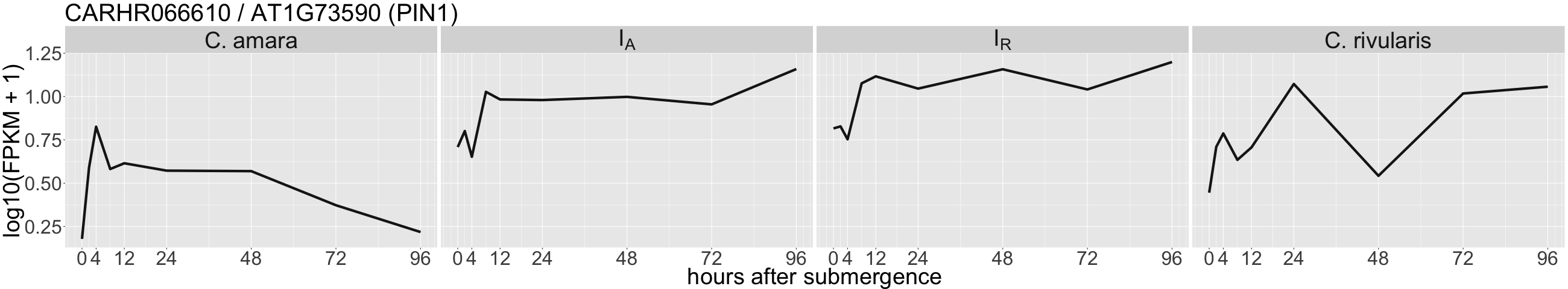

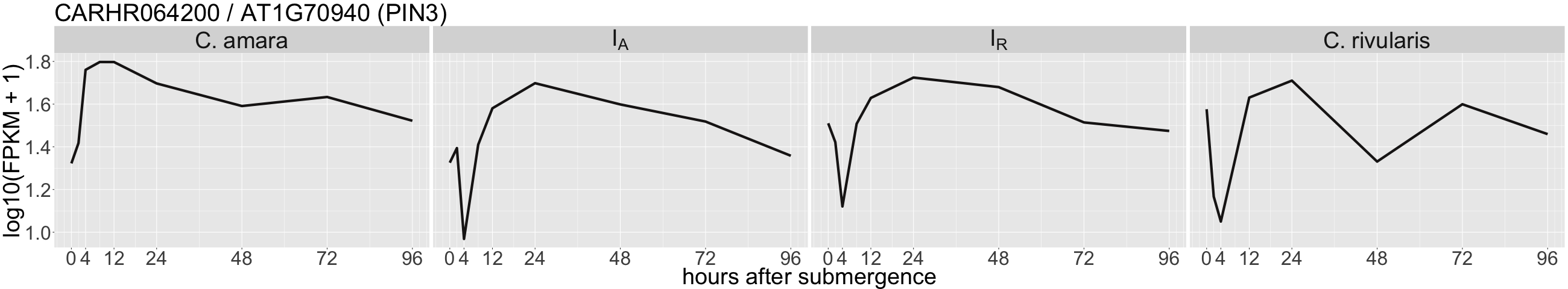

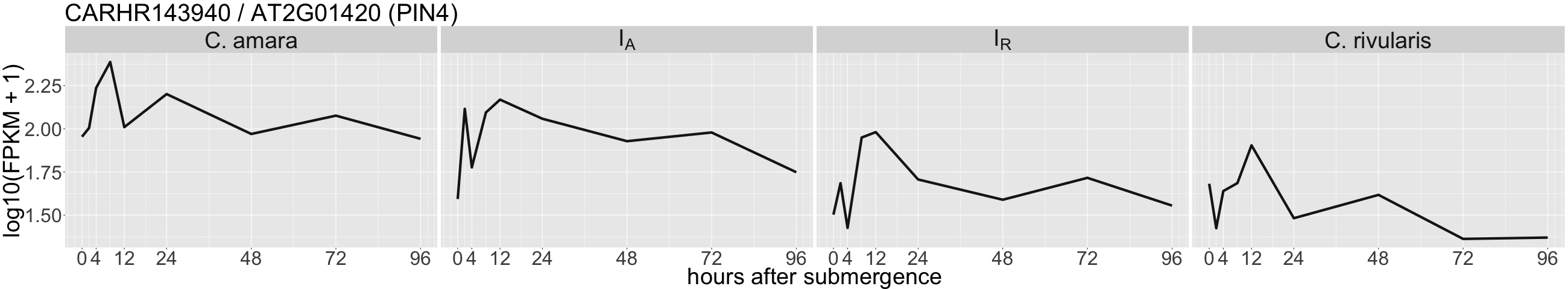

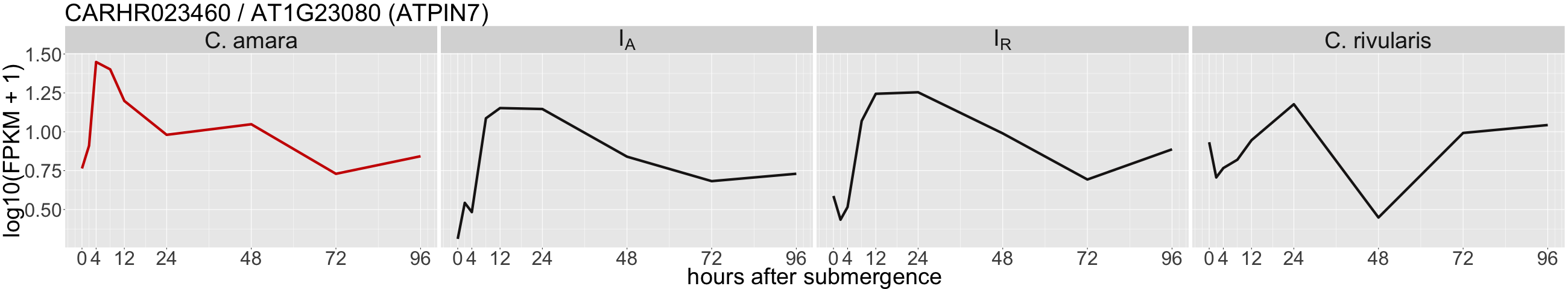

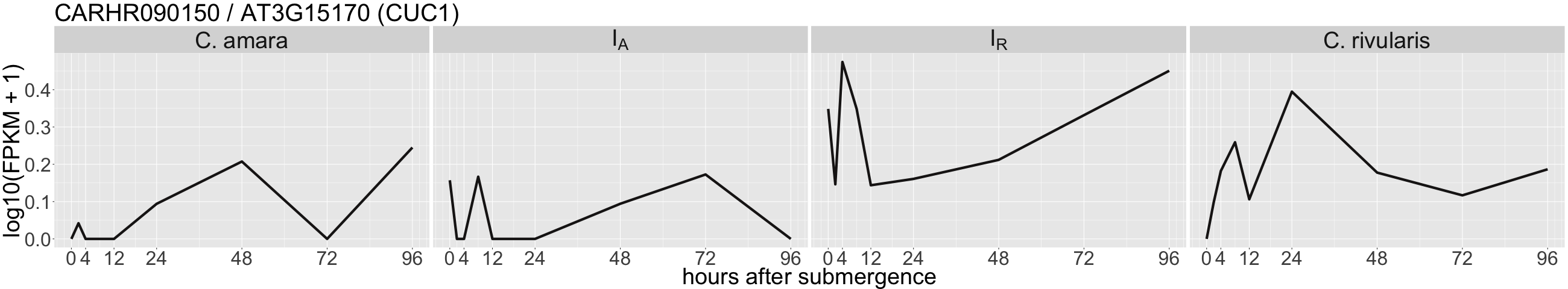

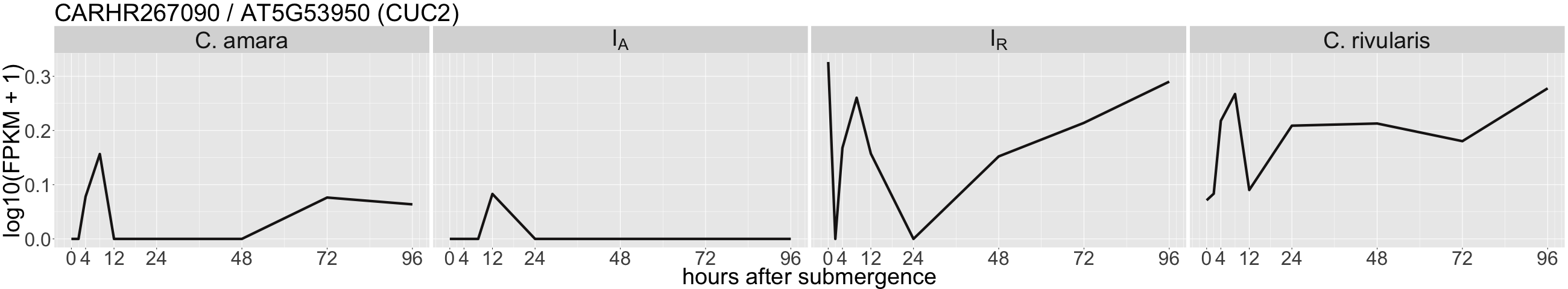

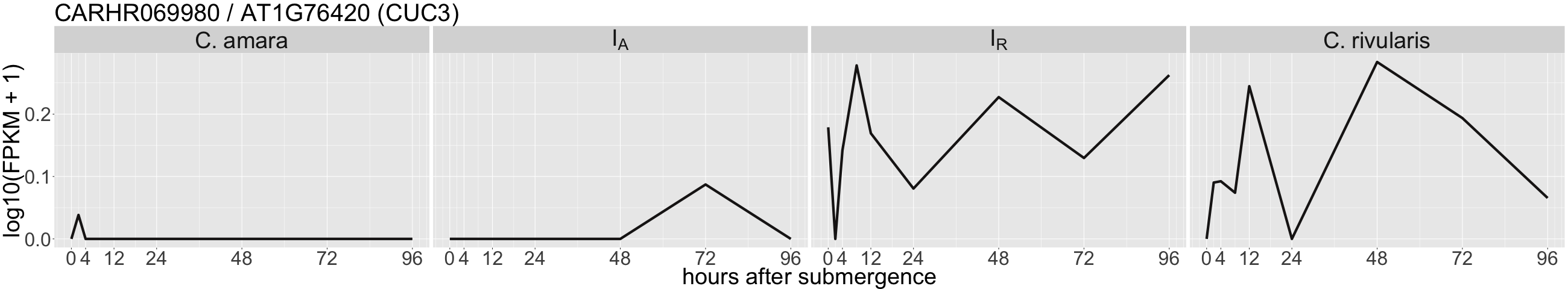

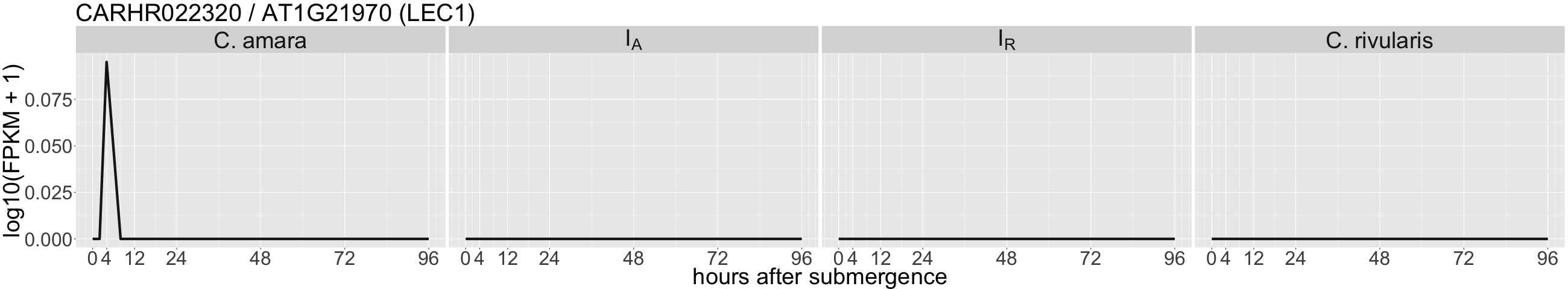

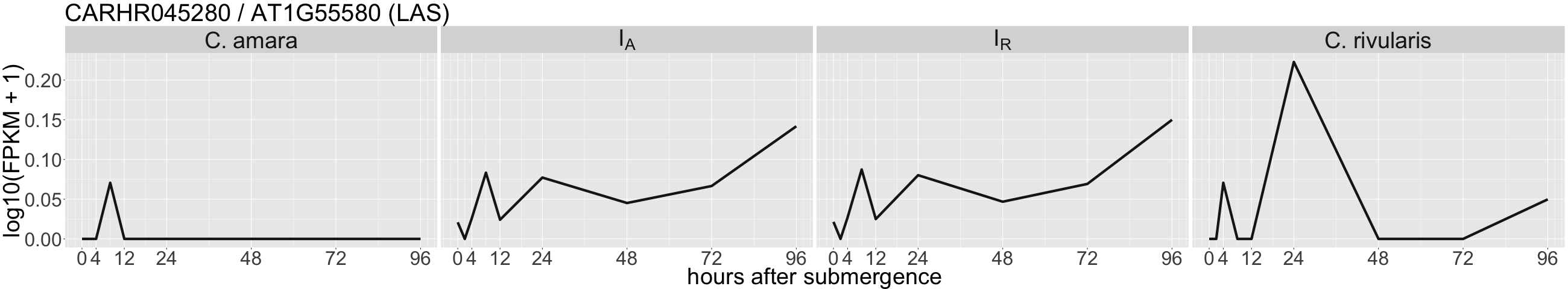

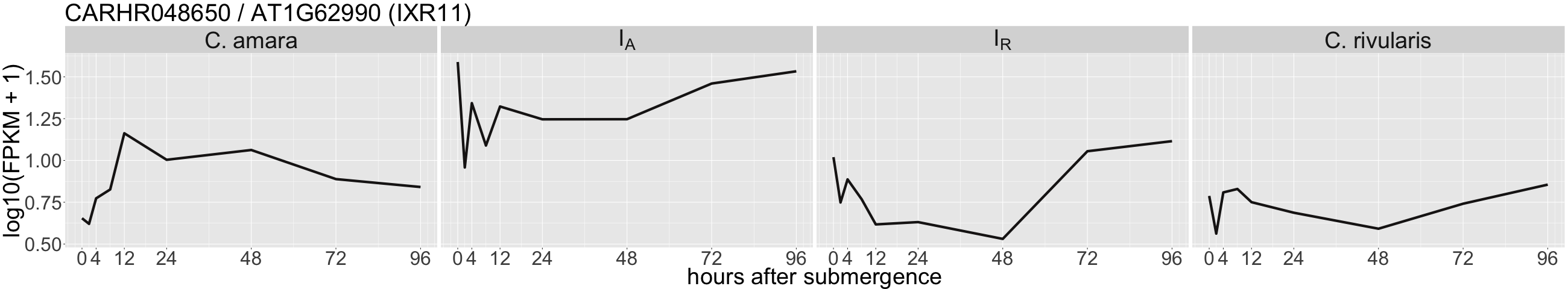

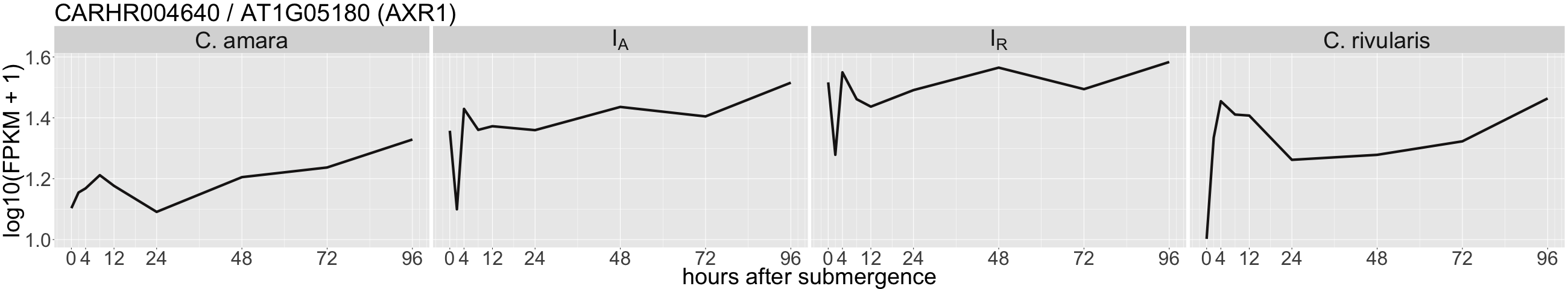

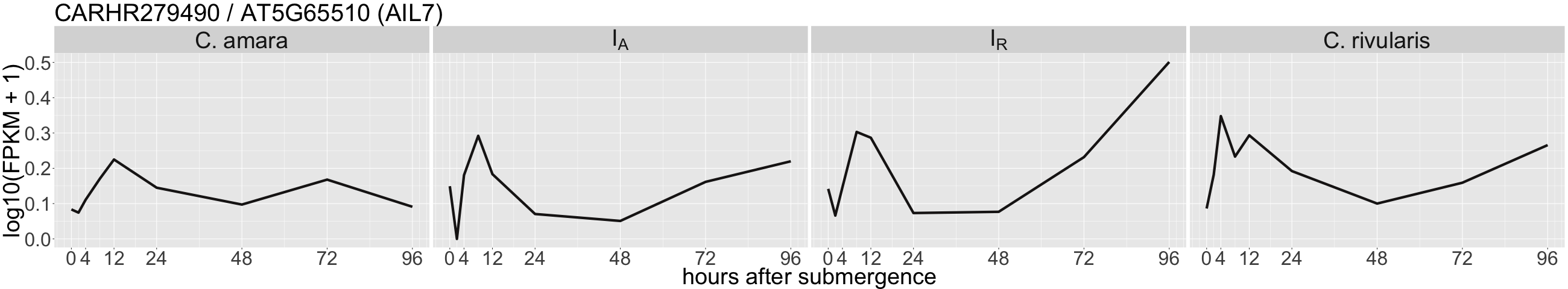

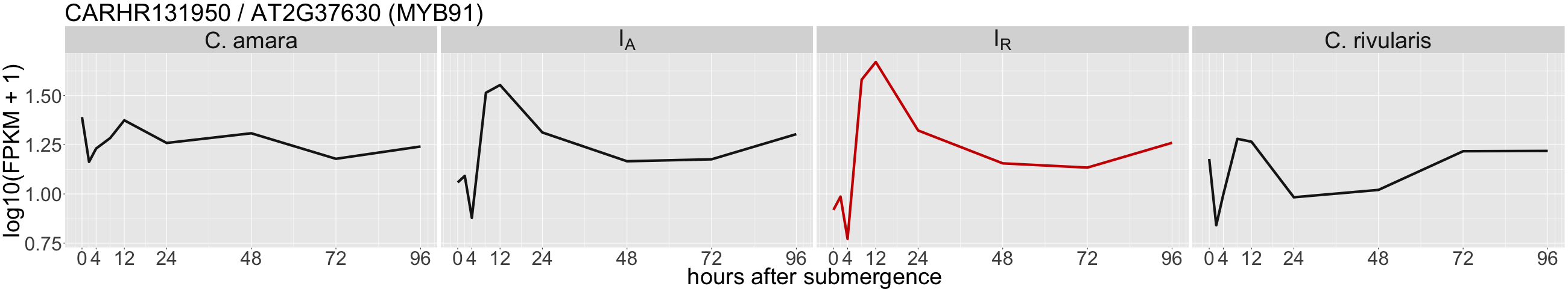

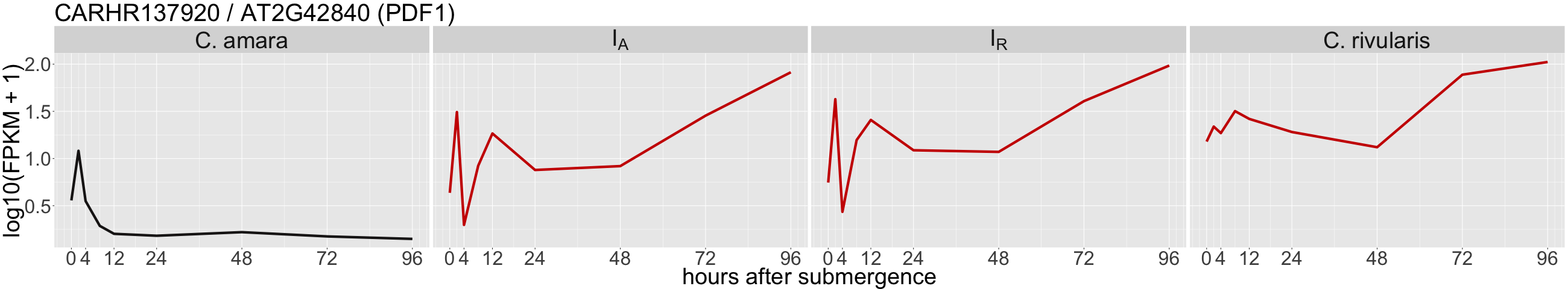

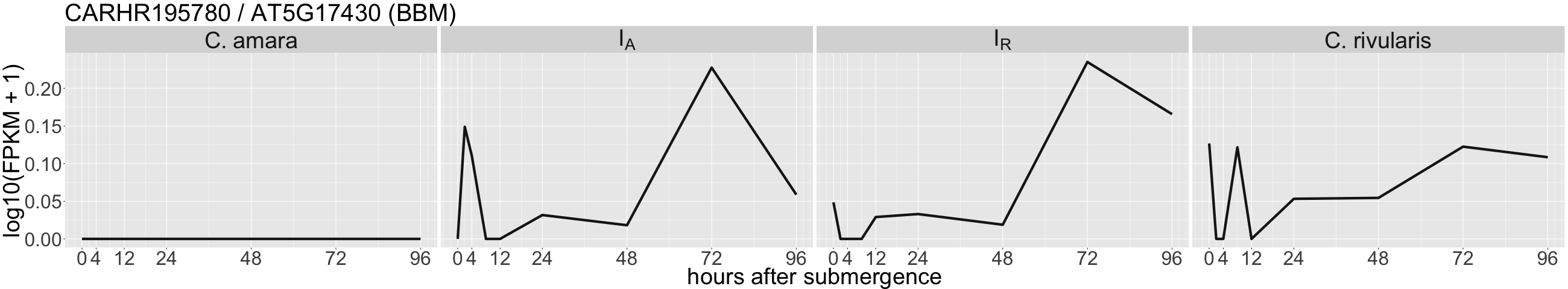

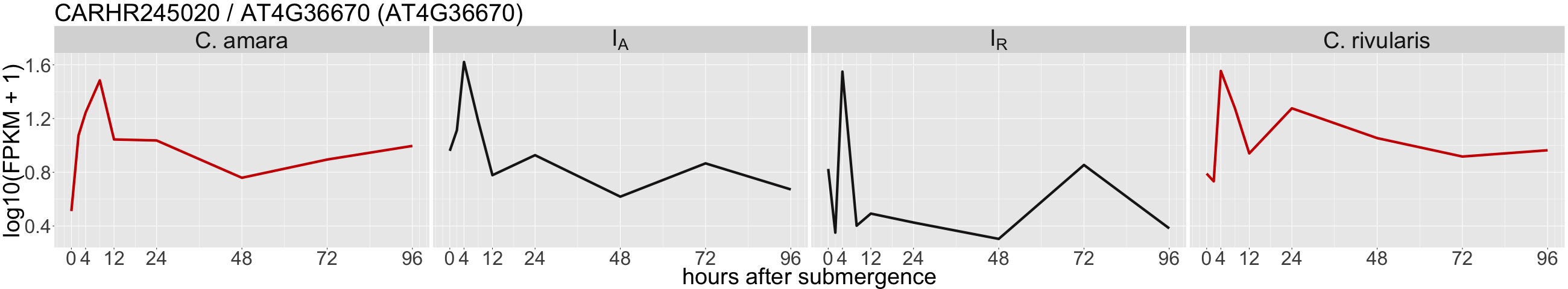

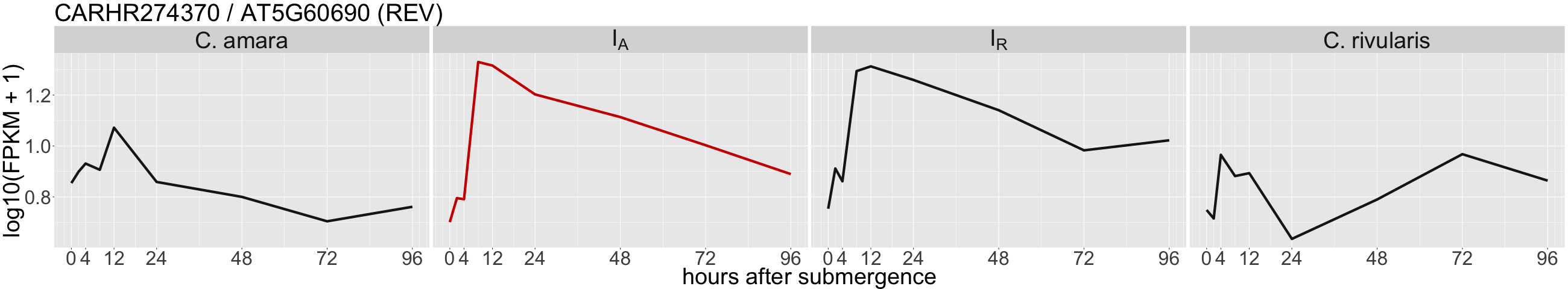


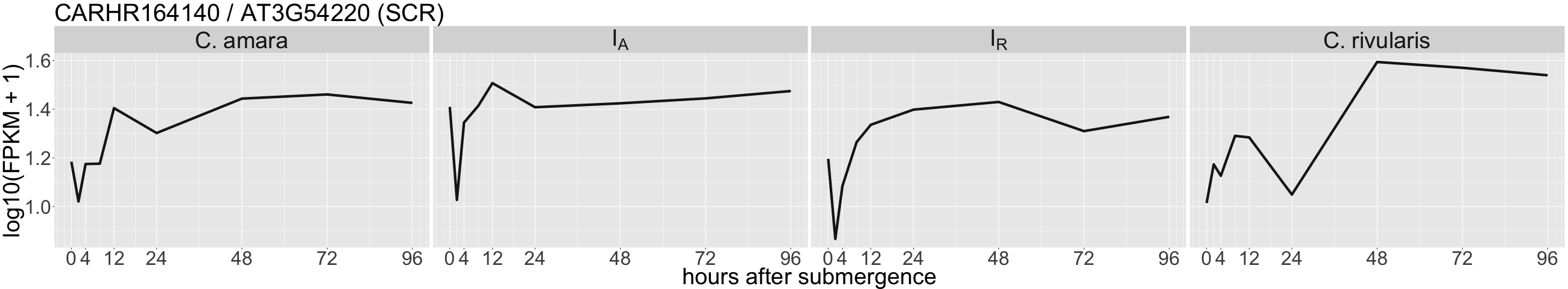


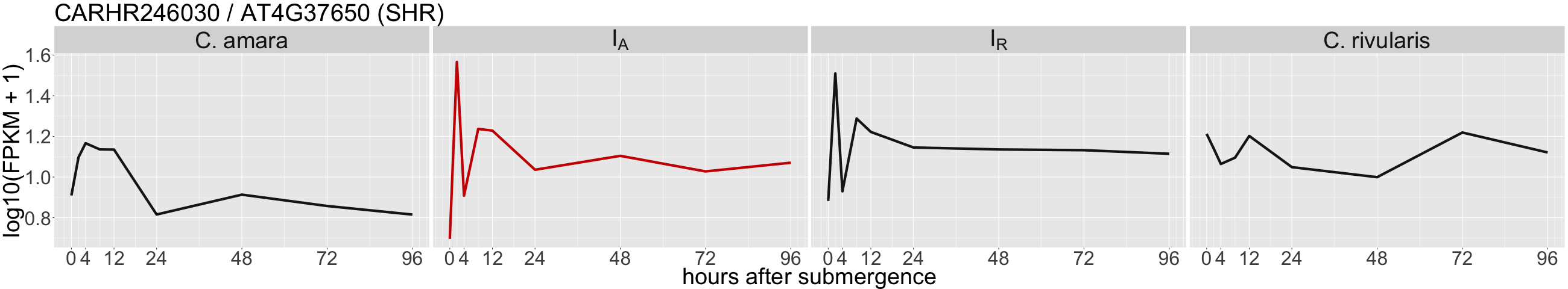


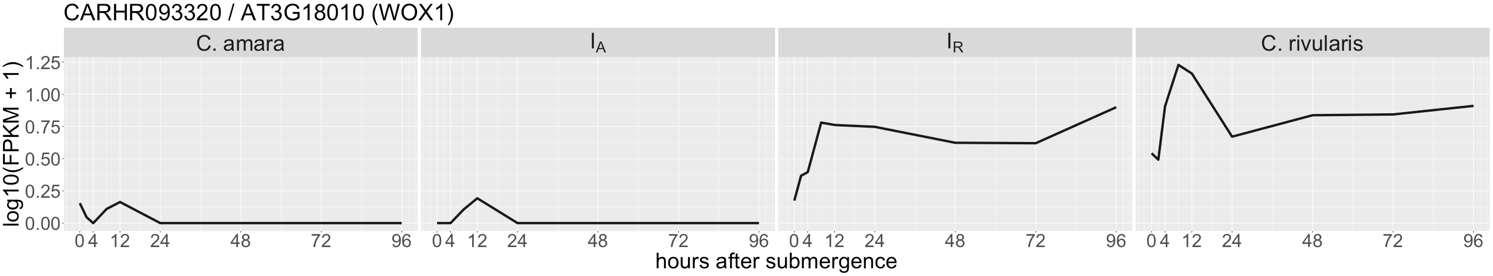


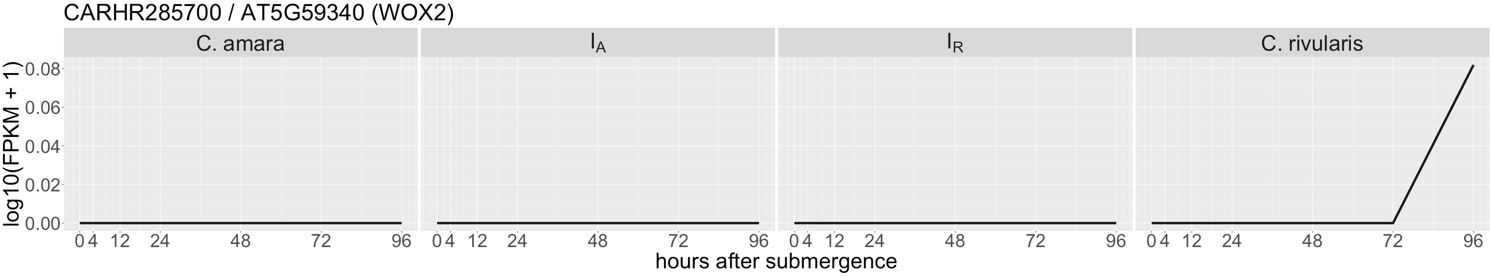


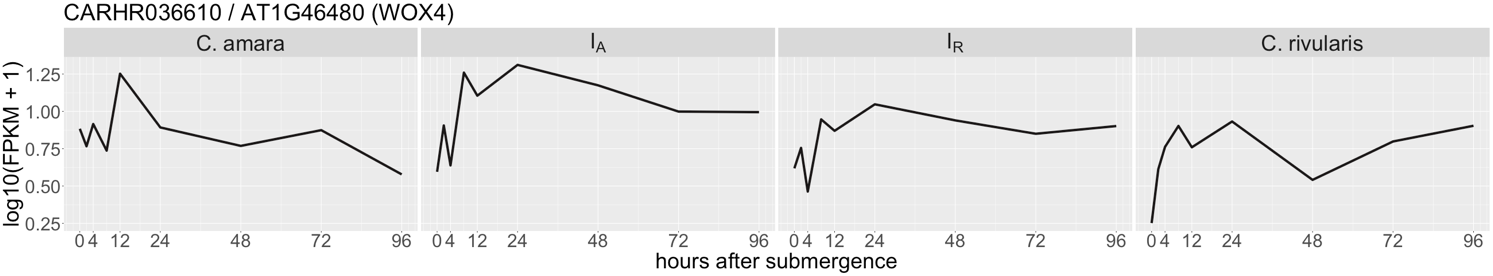


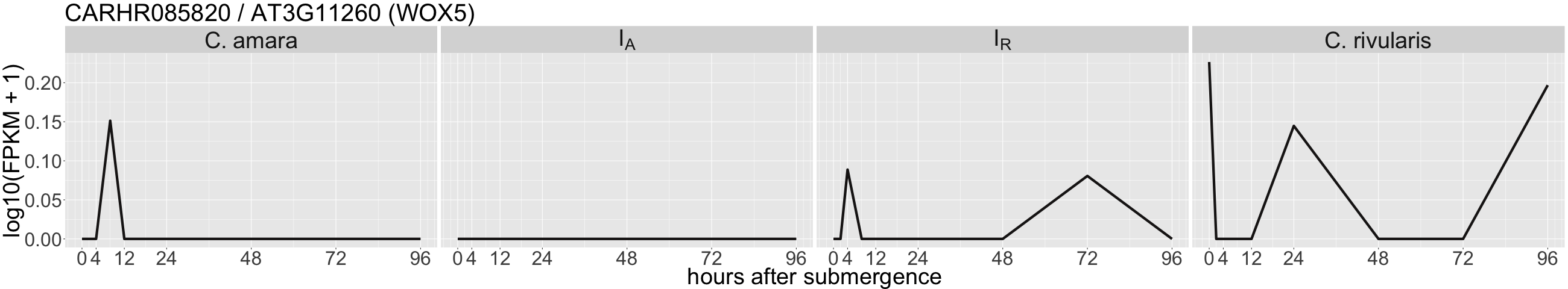


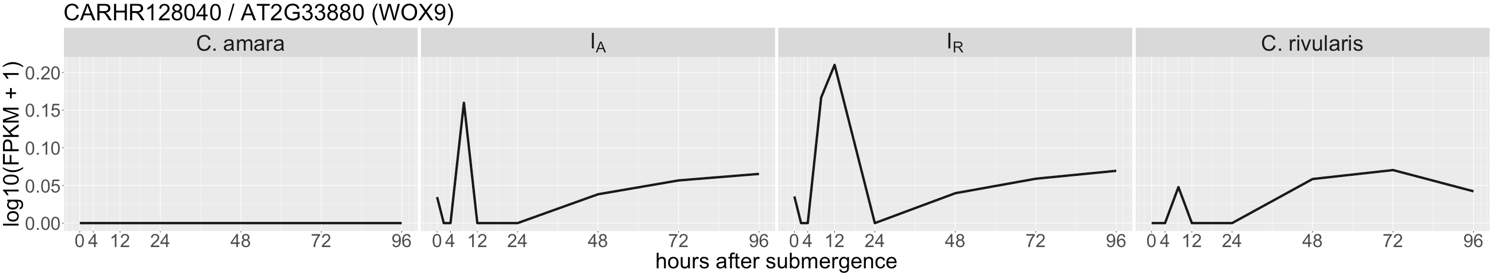


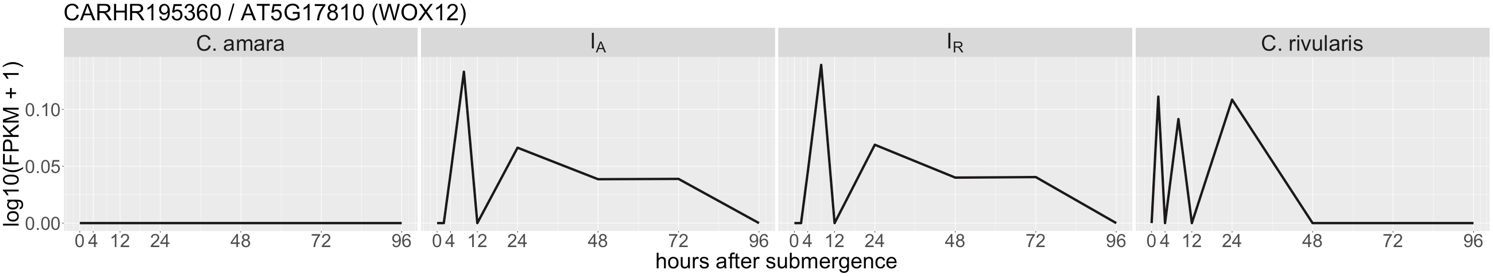


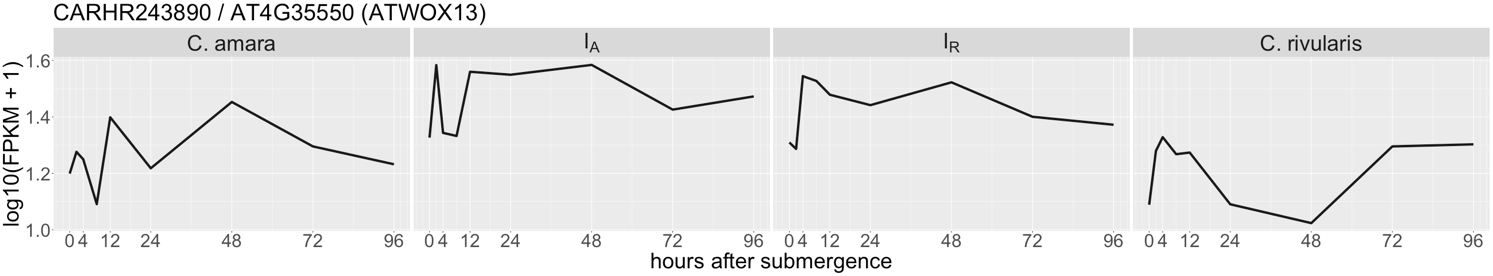


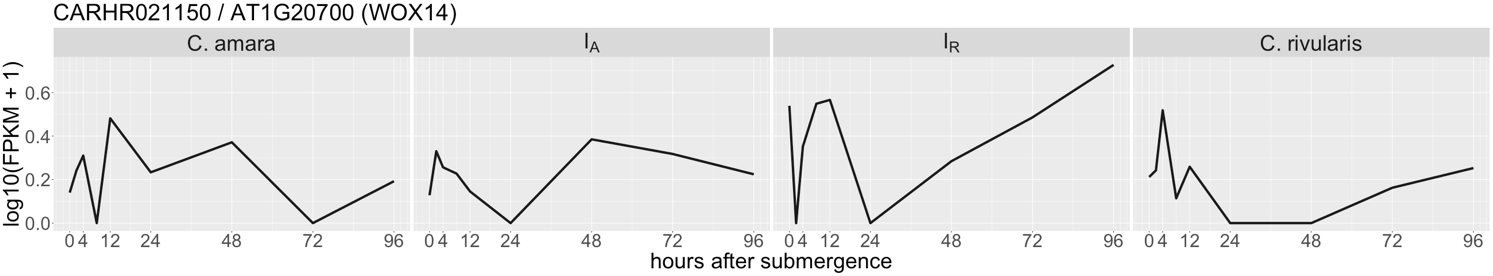


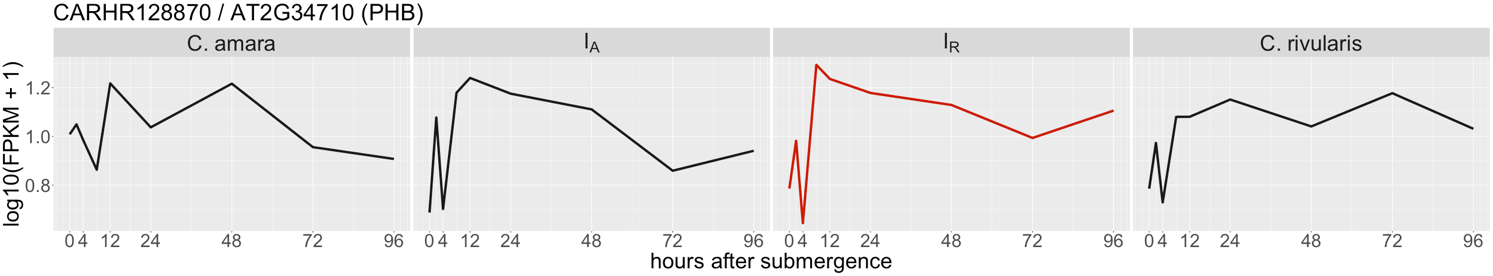


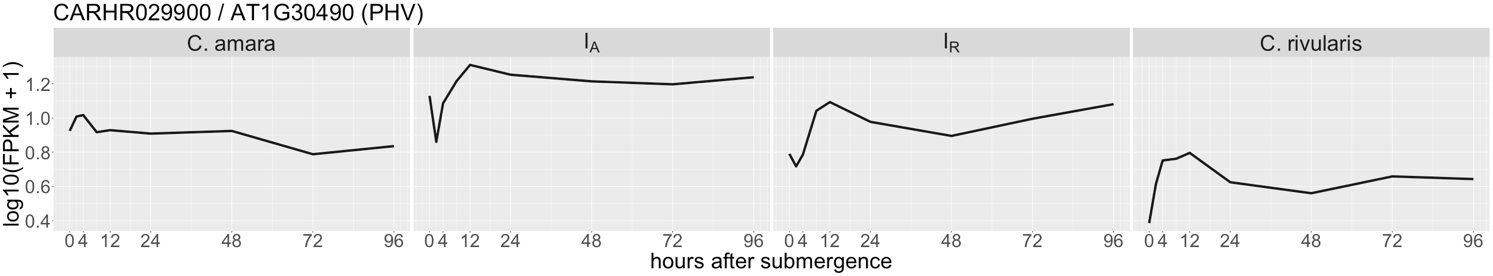


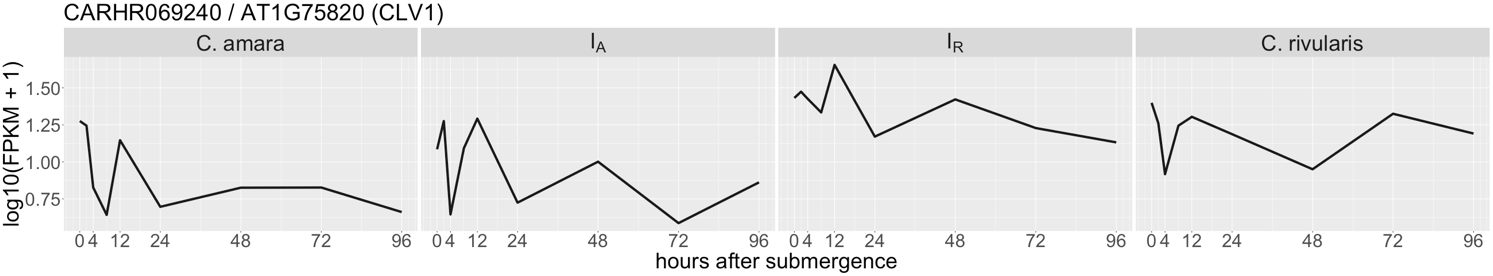


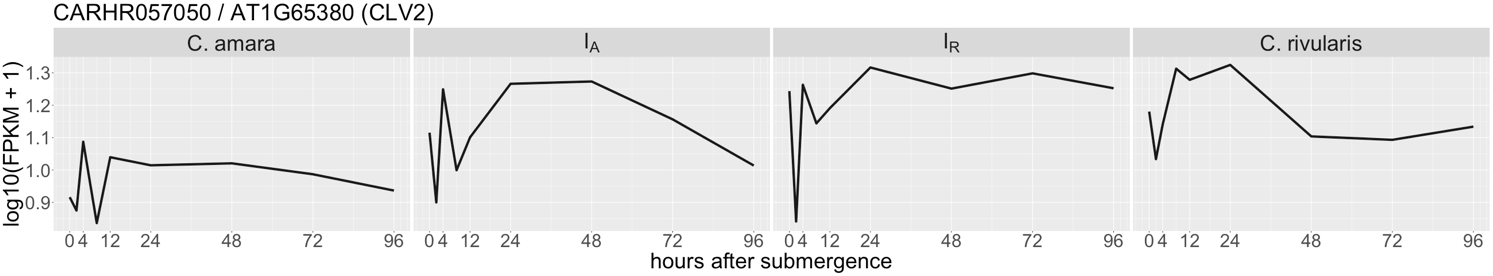


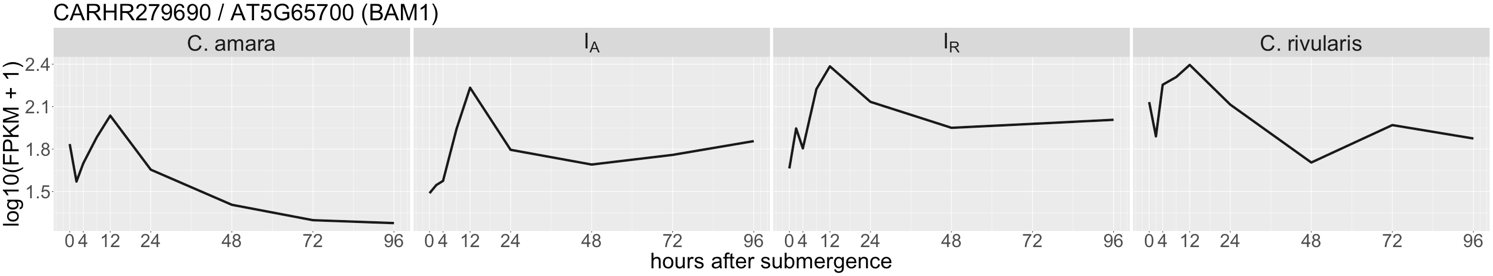


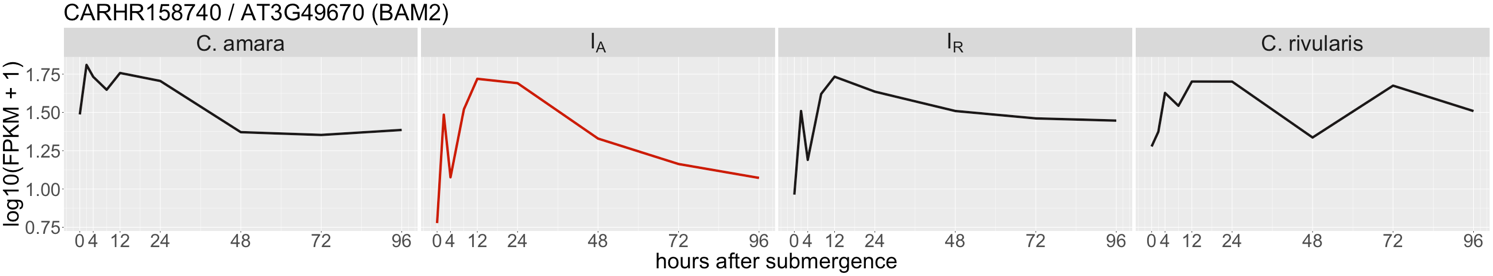


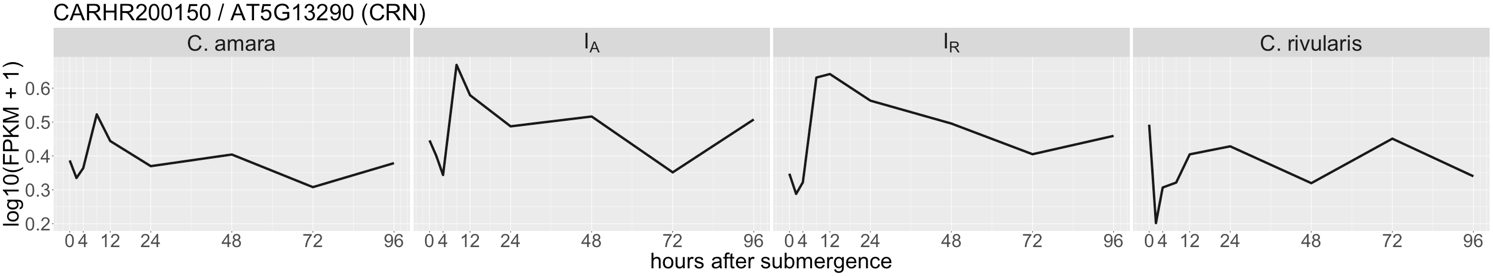

Supplement: Supplementary Figure 1 — Leaf vivipary in a representative leaf of C. rivularis. Anew plantlet was first visible 96 hours after submergence (circled, shown in close-up below). Plantlets initiated from dormant shoot meristems (shoot meristem with visible leaf shown). Shoot growth was detected 8 hours after submergence, followed by root initiation (16 hours). The shoot and root poles appeared to fuse (72 hours), followed by shoot and root growth to produce a plantlet (96 hours). [file Data_Sheet_1.zip › Supplementary_Figures_and_Tables.docx]
